# Photobiomodulation Therapy to Treat Snakebites Caused by *Bothrops atrox*: A Randomized Clinical Trial

**DOI:** 10.1001/jamainternmed.2023.6538

**Published:** 2023-12-04

**Authors:** Érica da Silva Carvalho, Andrea Renata do Nascimento Souza, Dessana Francis Chehuan Melo, Altair Seabra de Farias, Bruna Barbosa de Oliveira Macedo, Marco Aurélio Sartim, Mariela Costa Caggy, Beatriz de Alcântara Rodrigues, Gabriela Salini Ribeiro, Heloísa Nunes Reis, Felipe Queiroz Araújo, Iran Mendonça da Silva, André Sachett, Vanderson de Souza Sampaio, Antônio Alcirley da Silva Balieiro, Stella Regina Zamuner, João Ricardo Nickenig Vissoci, Lioney Nobre Cabral, Wuelton Marcelo Monteiro, Jacqueline de Almeida Gonçalves Sachett

**Affiliations:** 1 School of Health Sciences, Universidade do Estado do Amazonas, Manaus, Brazil; 2 Department of Teaching and Research, Fundação de Medicina Tropical Dr Heitor Vieira Dourado, Manaus, Brazil; 3 School of Medicine, Universidade Federal do Amazonas, Manaus, Brazil; 4 Universidade Nilton Lins, Manaus, Brazil; 5 Instituto Leônidas e Maria Deane - ILMD/Fiocruz, Manaus, Brazil; 6 Universidade Nove de Julho, Uninove, São Paulo, Brazil; 7 Department of Emergency Medicine and Duke Global Health Institute (DGHI), Duke University, Durham, North Carolina; 8 Department of Teaching and Research, Fundação Alfredo da Matta, Manaus, Brazil

## Abstract

**Importance:**

*Bothrops* venom acts almost immediately at the bite site and causes tissue damage.

**Objective:**

To investigate the feasibility and explore the safety and efficacy of low-level laser therapy (LLLT) in reducing the local manifestations of *B atrox* envenomations.

**Design, Setting, and Participants:**

This was a double-blind randomized clinical trial conducted at Fundação de Medicina Tropical Doutor Heitor Vieira Dourado, in Manaus, Brazil. A total of 60 adult participants were included from November 2020 to March 2022, with 30 in each group. Baseline characteristics on admission were similarly distributed between groups. Data analysis was performed from August to December 2022.

**Intervention:**

The intervention group received LLLT combined with regular antivenom treatment. The laser used was a gallium arsenide laser with 4 infrared laser emitters and 4 red laser emitters, 4 J/cm^2^ for 40 seconds at each application point.

**Main Outcomes and Measures:**

Feasibility was assessed by eligibility, recruitment, and retention rates; protocol fidelity; and patients’ acceptability. The primary efficacy outcome of this study was myolysis estimated by the value of creatine kinase (U/L) on the third day of follow-up. Secondary efficacy outcomes were (1) pain intensity, (2) circumference measurement ratio, (3) extent of edema, (4) difference between the bite site temperature and that of the contralateral limb, (5) need for the use of analgesics, (6) frequency of secondary infections, and (7) necrosis. These outcomes were measured 48 hours after admission. Disability assessment was carried out from 4 to 6 months after patients’ discharge. *P* values for outcomes were adjusted with Bonferroni correction.

**Results:**

A total of 60 patients (mean [SD] age, 43.2 [15.3] years; 8 female individuals [13%] and 52 male individuals [87%]) were included. The study was feasible, and patient retention and acceptability were high. Creatine kinase was significantly lower in the LLLT group (mean [SD], 163.7 [160.0] U/L) 48 hours after admission in relation to the comparator (412.4 [441.3] U/L) (*P* = .03). Mean (SD) pain intensity (2.9 [2.7] vs 5.0 [2.4]; *P* = .004), circumference measurement ratio (6.6% [6.6%] vs 17.1% [11.6%]; *P* < .001), and edema extent (25.8 [15.0] vs 40.1 [22.7] cm; *P* = .002) were significantly lower in the LLLT group in relation to the comparator. No difference was observed between the groups regarding the mean difference between the bite site temperature and the contralateral limb. Secondary infections, necrosis, disability outcomes, and the frequency of need for analgesics were similar in both groups. No adverse event was observed.

**Conclusions and Relevance:**

The data from this randomized clinical trial suggest that the use of LLLT was feasible and safe in a hospital setting and effective in reducing muscle damage and the local inflammatory process caused by *B atrox* envenomations.

**Trial Registration:**

Brazilian Registry of Clinical Trials Identifier: RBR-4qw4vf

## Key Points

**Question:**

What is the feasibility, safety, and efficacy of low-level laser therapy (LLLT) in reducing local manifestations of *Bothrops atrox* envenomations?

**Findings:**

In this double-blind randomized clinical trial that included 60 adults, the findings show that LLLT use was feasible and effective in reducing myonecrosis and the local inflammatory process caused by *B atrox* envenomations.

**Meaning:**

Results support the use of LLLT in combination with antivenom to treat snakebites.

## Teaser

This randomized clinical trial investigates the feasibility, safety, and efficacy of low-level laser therapy in reducing the local manifestations of *Bothrops atrox* envenomations.

## Introduction

Snakebite envenomations (SBEs) are considered a “disease of poverty,”^1^ as they mainly affect people living in impoverished rural communities in sub-Saharan Africa, Asia, Latin America, and Oceania.^2,3,4^ According to the World Health Organization, SBEs are part of the group of neglected tropical diseases^5^ and are responsible for significant socioeconomic effects in regions where there is a high incidence.^6,7^ In Latin America, the Amazon region has the highest incidence of SBEs, with about 50 cases per 100 000 inhabitants per year.^8^ *Bothrops atrox* is the most prevalent venomous snake in the Brazilian Amazon and causes 90% of the SBEs in the region.^9^ This species inhabits mostly forests, although it may occasionally be found in agricultural and urban environments.^10^

*B atrox* venom is composed of a complex mixture of biologically active molecules, including metalloproteinase as the main toxin family, followed by phospholipase A_2_, serine proteinases, cysteine-rich secretory proteins, L-amino acid oxidases, and C-type lectin-like toxins.^11,12^ These enzymatic components have predominantly proteolytic, coagulant, and hemorrhagic actions, which are responsible for the local and systemic events.^7,11,13^ Regarding local effects, *B atrox* envenomations lead to tissue damage, which is caused by intravascular coagulation, rupture of blood capillary vessels, and digestion of the extracellular matrix.^14,15^ These effects manifest clinically in signs and symptoms such as pain, swelling, regional lymphadenopathy, ecchymosis, blistering, and necrosis.^10^ Severe cases can progress to compartment syndrome, atrophy, loss of limb function, and even amputation.^16,17^

Antivenom is the only evidence-based treatment available for *Bothrops* snakebites.^18^ In vivo, *Bothrops* antivenom neutralizes the bleeding and lethality induced by the venom of *B atrox*.^19^ However, the efficacy of antivenom in reducing local tissue damage has been shown to be limited.^20,21^ *Bothrops* venom acts immediately at the bite site and has long-lasting local effects due to its activation of endogenous pathways that promote tissue damage,^14^ even after adequate antivenom treatment (AVT).^15^ In this regard, there is an urgent need to find complementary therapeutic approaches that can rapidly be deployed at the envenomation site to prevent severe local complications.

In in vitro and in vivo studies, photobiomodulation therapy (PBMT; low-level laser or light-emitting diode) has proved to be a promising supporting tool for treating the local effects induced by snake venoms.^22,23,24^ The biological effects that PBMT provokes in the tissues result in analgesic, anti-inflammatory, and healing actions.^25^ Low-level, laser-accelerated tissue repair modulates the immune response and provides a potentially useful resource to be used in association with antivenom.^26^ In animal models, PBMT has shown potential efficacy in attenuating the local effects of *Bothrops* envenomations, namely myonecrosis.^23,27,28^ Furthermore, PBMT reduces oxidative stress and enhances the phagocytosis capacity of macrophages.^29,30^

Evidence on SBE wound-related outcomes is scarcely reported, and specific treatment options for local tissue damage have not yet been studied in a clinical trial setting.^31^ While PBTM has been studied in other disease groups, in SBEs, this is the first time, to our knowledge, that PBTM has been tested in an SBE population. This study aims to investigate the feasibility and explore the safety and efficacy of low-level laser therapy (LLLT) in reducing the local manifestations of *B atrox* envenomations.

## Methods

### Study Design and Setting

This was a double-blind, 2-parallel-arm, 1:1 allocation ratio, randomized clinical trial to investigate the feasibility of PBMT using LLLT combined with the regular AVT compared to only AVT in the recovery of local inflammatory signs and symptoms (pain, edema, and temperature) and myolysis caused by *Bothrops* snakebites (trial protocol in Supplement 1). The clinical trial was performed at the Fundação de Medicina Tropical Doutor Heitor Vieira Dourado (FMT-HVD), in Manaus, Brazilian Amazon, from May 2020 to March 2022. This hospital is a reference hospital in the Amazonas state for treatment of snakebites. In Manaus, FMT-HVD is the only hospital unit that administers snakebite AVT. On admission, *Bothrops* snakebites were diagnosed based on clinical and epidemiological characteristics and, when the patient brought the snake responsible for the envenomation, by the identification of the perpetrating snake, which was done by a trained biologist using a validated taxonomic key for the study region.^32^ Data were collected at the bedside during the hospital stay.

This study was registered in the Brazilian Clinical Trials Registry (ReBec): RBR-4qw4vf and UTN No. U1111-1244-4898. Ethical approval was obtained from the Ethics Review Board of FMT-HVD (approval No. 3.639.449/2019). Written informed consent was obtained from all participants prior to randomization and only after a detailed explanation of the study protocol. All the interventions were provided at no cost to the patients. This study followed the Consolidated Standards of Reporting Trials (CONSORT) reporting guideline.

### Sample Calculation

Calculation of the sample size was designed to estimate the standard deviation of the targeted primary outcome, with 90% power and 2-tailed 5% significance. Thus, assuming the rationale of the noncentral *t*-distribution approach, a sample size of 28 participants per treatment arm was enough to estimate the standard deviation assuming a standardized small (0.2) effect size.^33^ To ensure minimal effects of loss to follow-up, we added 2 participants in each arm. Thus, a sample size of 60 participants was obtained, with 30 in the intervention group (LLLT plus AVT) and 30 in the comparator group (standard AVT).

### Participants, Recruitment, Randomization, and Blinding

Participants were recruited and consent was obtained by a member of the research team (E.S.C.), after being identified and referred by the attending clinician. Eligible patients were male or female individuals 18 years and older, with a clinical-epidemiological diagnosis of a *Bothrops* (pit viper) envenomation presenting at FMT-HVD. Exclusion criteria consisted of patients who were admitted to the hospital more than 24 hours after the bite, those who had had AVT administered at another health facility prior to hospital admission, those who had a secondary infection, pregnant individuals, patients with history of diabetes, those with ulcers or other types of wounds by other causes in the affected limb, immunocompromised patients, those with bites near the eyes, the presence of tattoos in the area of LLLT application, or those with dry bites, ie, cases with fang marks but no signs of envenomation.^34^

Eligible patients were randomly assigned (1:1) by a computer-generated randomization schedule to receive LLLT plus AVT (intervention group) or the regular AVT (comparator group). Patients and the research, clinical, and laboratory teams were blinded for treatment assignment.

### Intervention

AVT was given to all patients in the intervention and comparator groups according to clinical severity. *Bothrops* or *Bothrops-Lachesis* AVT was administered according to the guidelines of the Brazilian Ministry of Health,^18^ and cases were classified as (1) mild: local pain, swelling, and bruising; (2) moderate: local manifestations without necrosis and minor systemic signs (coagulopathy and bleeding, no shock); or (3) severe: life-threatening snakebite, with severe bleeding, hypotension, shock, and/or acute kidney failure.^18^ AVT was given 30 minutes after premedication with intravenous hydrocortisone (500 mg), intravenous cimetidine (300 mg), and oral dexchlorpheniramine (5 mg) (standardized according to local guidelines). Before administration, AVT vials were diluted in 100 mL of 0.9% saline. AVT administration time ranged from 30 to 45 minutes.

Both groups underwent the same local wound care, ie, daily cleaning with 0.9% saline. Per the hospital protocol, for pain, 1 g of intravenous metamizole every 6 hours was given on demand. Persistent intense pain was treated with 100 mg of intravenous tramadol.

Per current usual care, the affected limb was treated in the most comfortable position, according to patient preferences. When present, blisters were aspirated, necrotic tissue was surgically debrided, abscesses were drained, and antibiotic treatment was given accordingly.

#### Intervention Group

LLLT was used combined with regular AVT. The e-Light IRL (SN 0608) gallium arsenide device (DMC Equipamentos) was used in the LLLT (eFigure 1 in Supplement 2). This laser device has 8 light emitters, 4 of which emit red light (660 nm) and 4 emit infrared light (808 nm), with a potency of 100 mW. In this study, each emitter radiated 4 J/cm^2^ for 40 seconds at each application point. This dosage was selected for its efficacy demonstrated in mice to reduce myonecrosis induced by *Bothrops jararacussu* snake venom.^23^ This treatment regimen was defined to fall below the safety margins of laser intensity to avoid adverse reaction of the intervention.^35^ The delimitation of the area in which LLLT was applied was carried out with the help of a FLIR C2 thermographic camera (Teledyne FLIR). Thermography is a clinical tool that uses infrared imaging to quantify body surface temperature, capturing the emitted radiation and producing a high-resolution digital image.^36^ This image is made visible through a color scale in which the warmest areas are represented in white or red, while the coldest areas appear in blue or black. The venom induces local inflammatory reactions, and, in thermography, a white area represents where the inflammation is most intense.^37^ In this study, LLLT was applied to the white areas (eFigure 2 in Supplement 2). LLLT was performed once a day for 3 consecutive days, with the first application made on the day of hospital admission, 30 minutes after the AVT, with an interval of 24 hours between applications. The patients and the researcher responsible for LLLT wore proper safety glasses for laser light during the procedure to avoid eye damage. The development of a secondary bacterial infection was a criterion for withdrawing the patient from the study.

#### Comparator Group

This group received the same attention as the intervention group, but with the laser device turned off. The use of safety glasses by the patients in this group prevented them from realizing that the equipment was not working during the procedure, thus ensuring blinding.

The researcher (E.S.C.) who applied the laser therapy was not blinded to this procedure. However, all outcome assessments were performed by a single, blind assessor (G.S.R.). As a result, blinding was ensured when obtaining these measurements.

### Baseline Data

After inclusion of the patient, demographic and epidemiological data were collected using a standardized form. Baseline data included information on sex, age (in years), area of occurrence (urban or rural), anatomical site of the bite, time elapsed from bite to medical assistance (in hours), walking after bite (in minutes), previous history of snakebite, and preadmission treatments (use of topical or oral medicines, use of tourniquet and other procedures). A detailed clinical and laboratorial characterization was performed on admission and 24 and 48 hours after admission, before LLLT. Presence of local bleeding, lymphadenitis, and necrosis was assessed. Any systemic signs and symptoms, such as systemic bleeding, acute kidney failure, headache, dizziness, and vomiting, were recorded. Pain assessment was made using a numerical rating scale, with values ranging from 1 to 10^38^; pain was further classified as absent (rate 0), mild (rated from 1 to 3), moderate (rated from 4 to 7), and intense (rated from 8 to 10). Edema was assessed with a tape measure to obtain the circumference of the affected limb over the bite site and on the contralateral limb; the ratio between the 2 measures was calculated. Circumference was measured at the level of the fang marks. The extent of edema in the affected limb was also evaluated by measuring the distance between the distal and proximal points showing swelling. Bite site temperature was measured using an infrared digital thermometer (Color Check AC322); the difference between the bite site temperature and that of the contralateral limb was calculated. Immediately after clinical examination, 15 mL of blood was taken for laboratorial analysis. Tests included leukocyte count (cells/μL; to convert to ×10^9^/L, multiply by 0.001), platelet count (number/μL; to convert to ×10^9^/L, multiply by 0.001), hemoglobin (mg/dL; to convert to g/L, multiply by 0.01), creatine kinase (U/L; to convert to μkat/L, multiply by 0.0167), creatinine (mg/dL; to convert to μmol/L, multiply by 88.4), urea (mg/dL; to convert to mmol/L, multiply by 0.357), Lee-White clotting time (in minutes), prothrombin time (in seconds), and C-reactive protein (mg/dL; to convert to mg/L, multiply by 10). Disability assessment was performed by phone interview 4 to 6 months after patients’ discharge, using the World Health Organization Disability Assessment Schedule 2.0 (WHODAS 2.0), with values ranging from 1 (no difficulty) to 5 (extreme difficulty).^39^ All demographic, clinical, and laboratory information was collected through a standardized clinical registration form (REDCap, Vanderbilt University).

### Feasibility Outcomes

We obtained patient eligibility rates, recruitment rates, and retention rates. Retention rate was calculated at 48 hours after admission, which is the time at which the primary outcome was measured, and at 4 to 6 months after patients’ discharge using the WHODAS 2.0, to determine the long-term disability outcome in this study.

Data on the fidelity in the treatment protocol were obtained by measuring the ratio of interventions that needed adaptations/changes over the total number of interventions. Our goal was to verify whether fidelity was a potential issue in this trial.

For feasibility, we measured the trial design feasibility via deviations from the evaluation protocol regarding the blinding and data collections. Protocol deviations were measured by the ratio of participants with any deviation from the protocol/breaking of blinding to the total number of patients.

Patient acceptability was assessed by asking 2 questions: (1) Did you have any discomfort during the LLLT application? (2) Do you agree that we may perform another LLLT application?

### Safety Outcomes

Adverse events, such as itching, skin redness, changes in skin pigmentation, bruising, scarring, peeling, skin depression, and textural and thickness changes, were described in both arms, during the follow-up.

### Efficacy Outcomes

#### Primary Outcomes

The primary efficacy outcome was myolysis estimated by the value of creatine kinase 48 hours after admission. Additionally, the time from admission to reduction of creatine kinase activity by 50% was also calculated, in a follow-up of 48 hours.

#### Secondary Outcomes

The exploratory efficacy outcomes were (1) pain intensity as measured by a numerical scale and time from admission to reduction of pain intensity by 50%, at 48 hours after admission; (2) the circumference measurement ratio (circumference of the affected limb/circumference of the contralateral limb; expressed as a percentage) and time from admission to reduction of circumference measurement ratio by 50%, at 48 hours after admission; (3) extent of edema in the affected limb, by measuring the distance between the distal and proximal points showing swelling (in centimeters), and time from admission to the reduction of the extent of edema by 50%, at 48 hours after admission; (4) difference between the bite site temperature and that of the contralateral limb (in °C) and time from admission to this reduction by 50%, at 48 hours after admission; (5) need for the use of analgesics in a follow-up of 48 hours; (6) frequency of secondary infections in a follow-up of 48 hours; (7) necrosis in a follow-up of 48 hours; and (8) disability assessment from 4 to 6 months after patients’ discharge using the WHODAS 2.0. For the disability assessment, patients were contacted by phone, up to 3 times on consecutive days. Those who did not answer the calls were considered as being lost to follow-up.

### Statistical Analysis

Feasibility outcomes were analyzed using descriptive statistics as proportions. Trial outcomes were reported as means and standard deviations. To evaluate the efficacy, we report the differences between the 2 groups. Mean values of creatine kinase activity, pain intensity, circumference measurement ratio, extent of edema, and difference between the bite site temperature and that of the contralateral limb were compared using the *t* test for independent samples, at 48 hours after admission, with Bonferroni correction applied. In addition, a Kaplan-Meier survival analysis with a log-rank test was performed to detect differences in the time elapsed from admission to the day of reduction by 50% in creatine kinase activity, pain intensity, circumference measurement ratio, extent of edema, and difference between the bite site temperature and that of the contralateral limb between the intervention and comparator groups, at 2 points—24 and 48 hours after admission. The comparison of the frequency of use of analgesics, secondary infections, and necrosis in a follow-up of 48 hours between the intervention and comparator groups was made using the χ^2^ or Fisher exact test; odds ratios (ORs) with 95% CIs were obtained. The Kruskal-Wallis rank sum test was used to compare the disability scores between groups for each domain and for the summary score. Statistical analyses were performed in the R software in the IDE RStudio environment, version 4.1.2 (Posit PBC), and the significance level of the tests was *P* < .05.

## Results

### Characterization of the Participants

A total of 60 patients (mean [SD] age, 43.2 [15.3] years; 8 female individuals [13%] and 52 male individuals [87%]) were included. Epidemiological, clinical, and laboratory characteristics of the study participants are presented in Table 1. These characteristics were similarly distributed between the intervention and comparator groups on admission.

**Table 1.** Epidemiological, Clinical, and Laboratory Characteristics of the Study Participants

Variable	No. (%)		
	Total (N = 60)	Intervention (n = 30)^a^	Comparator (n = 30)^b^
Sex			
Female	8 (13)	6 (20)	2 (7)
Male	52 (87)	24 (80)	28 (93)
Rural area of occurrence	47 (78)	22 (73)	25 (83)
Age group, y			
18-30	15 (25)	6 (20)	9 (30)
31-40	12 (20)	5 (17)	7 (23)
41-50	14 (23)	9 (30)	5 (17)
51-60	9 (15)	5 (17)	4 (13)
>60	10 (17)	5 (17)	5 (17)
Bite site			
Hand	5 (8)	4 (13)	1 (3)
Foot	30 (50)	14 (47)	16 (53)
Leg	15 (25)	7 (23)	8 (27)
Arm	2 (3)	1 (3)	1 (3)
Toes	3 (5)	2 (7)	1 (3)
Fingers	5 (8)	2 (7)	3 (10)
Walking after bite, min			
No	21 (35)	12 (40)	9 (30)
5-9	7 (12)	3 (10)	4 (13)
10-29	19 (32)	11 (37)	8 (27)
30-59	9 (15)	2 (7)	7 (23)
≥60	4 (7)	2 (7)	2 (7)
Time to medical assistance, h			
0-3	33 (55)	19 (63)	14 (47)
4-6	14 (23)	5 (17)	9 (30)
7-12	4 (7)	2 (7)	2 (7)
13-24	9 (15)	4 (13)	5 (17)
Previous snakebite	10 (17)	6 (20)	4 (13)
Use of topical medicines	15 (25)	7 (23)	8 (27)
Use of oral medicines	16 (27)	8 (27)	8 (27)
Use of tourniquet	17 (28)	8 (27)	9 (30)
Severity classification^c^			
Mild	10 (17)	6 (20)	4 (17)
Moderate	38 (63)	19 (63)	19 (63)
Severe	12 (20)	5 (117)	7 (20)
Prescription of analgesics			
Intravenous metamizole, 1 g every 6 h	60 (100)	30 (100)	30 (100)
Intravenous tramadol, 100 mg	35 (58)	17 (57)	18 (60)
Local bleeding	22 (37)	13 (43)	9 (30)
Headache	26 (43)	12 (40)	14 (47)
Dizziness	2 (3)	2 (7)	0
Gingival bleeding	3 (5)	1 (3)	2 (7)
Vomiting	8 (13)	3 (10)	5 (17)
Nausea	14 (23)	5 (17)	9 (30)
Leukocytes, mean (SD), ×10^3^/mm^3^	12.7 (5.7)	12.4 (5.5)	13.1 (6.0)
Platelets, mean (SD), ×10^3^/mm^3^	254.1 (309.9)	305.9 (432.6)	202.3 (52.0)
Hemoglobin, mean (SD), g/dL	14.7 (1.5)	14.8 (1.4)	14.6 (1.6)
Creatinine, mean (SD), mg/dL	1.01 (0.30)	1.07 (0.24)	0.95 (0.35)
Urea, mean (SD), mg/dL	39 (16)	41 (16)	37 (15)
Lee-White clotting time			
Clottable	20 (33)	13 (43)	7 (23)
Unclottable	40 (67)	17 (57)	23 (77)
Prothrombin time			
Normal (≤14 s)	2 (3)	2 (7)	0
Abnormal (>14 s)	58 (97)	28 (93)	30 (100)
C-reactive protein increased	14 (23)	6 (20)	8 (27)

SI conversion factors: To convert leukocytes and platelets ×10^3^/mm^3^ to ×10^9^/L, multiply by 0.001; hemoglobin mg/dL to g/L, by 0.01; creatinine mg/dL to μmol/L, by 88.4; urea mg/dL to mmol/L, by 0.357.

^a^ Low-intensity laser therapy combined with the regular antivenom treatment.

^b^ Regular antivenom treatment.

^c^ According to the Brazilian Ministry of Health classification.

### Feasibility, Fidelity, and Safety Outcomes

**Figure 1.**
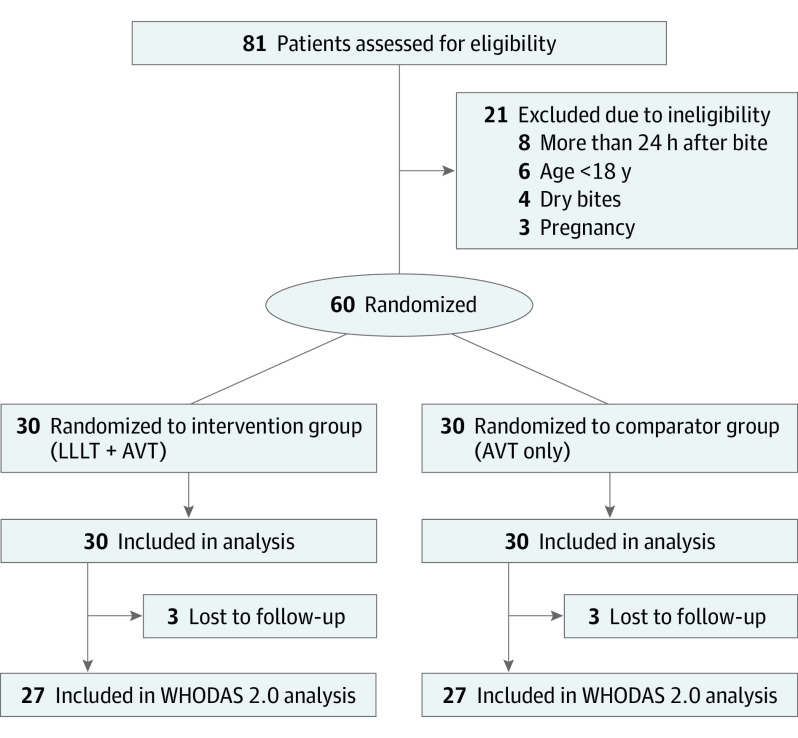
Flowchart for the Inclusion of Study Participants AVT indicates antivenom treatment; LLLT, low-level laser therapy; WHODAS 2.0, World Health Organization Disability Assessment Schedule 2.0.

A total of 81 patients were assessed for eligibility during the study period. Of this total, 60 (74%) met the inclusion criteria and were invited to participate in the study. The main reason for ineligibility was hospital admission after more than 24 hours since the snakebite. All 60 patients who met eligibility criteria consented to participate in the study and were randomized. Retention rate was 100% at 48 hours after admission, which is the time at which the primary outcome was measured, and 90% at 4 to 6 months after the patient’s discharge using the WHODAS 2.0; for this outcome, retention ratio was identical between the intervention and comparator groups (Figure 1). All patients who had a secondary bacterial infection developed this complication 48 hours after admission. Thus, no patient was withdrawn from the study due to the development of a secondary bacterial infection until the third LLLT application.

Fidelity was not an issue with this trial because none of the interventions needed adaptations/changes. As for feasibility, no protocol deviations were observed due to the protocol being broken in terms of blinding and data collection. No patient reported any discomfort during the LLLT application or expressed that they would like to withdraw from the study as a result of any procedure performed. Regarding safety outcomes, no adverse events were observed in either study arm.

### Efficacy Outcomes

#### Primary Outcome

Mean (SD) creatine kinase values 48 hours after admission were significantly lower in the LLLT group (163.7 [160.0] U/L) in relation to the comparator group (412.4 [441.3] U/L) (*P* = .03).

#### Secondary Outcomes

Mean (SD) pain intensity 48 hours after admission was significantly lower in the LLLT group (2.9 [2.7]) in relation to the comparator group (5.0 [2.4]) (*P* = .004). The mean (SD) circumference measurement ratio was significantly lower in the LLLT group (6.6% [6.6%]) in relation to the comparator group (17.1% [11.6%]) (*P* < .001). The mean (SD) edema extent was significantly lower in the LLLT group (25.8 [15.0] cm) in relation to the comparator group (40.1 [22.7] cm) (*P* = .002). No difference was observed between the 2 groups regarding the mean difference between the bite site temperature and the contralateral limb (Table 2).

**Table 2.** Efficacy Outcomes Results

Outcome	At admission			48 h		
	Mean (SD)		*P* value^a^	Mean (SD)		*P* value^a^
	Intervention (n = 30)^b^	Comparator (n = 30)^c^		Intervention (n = 30)^b^	Comparator (n = 30)^c^	
**Primary outcome**						
Creatine kinase, 48 h after admission, U/L	483.8 (659.7)	339.9 (269.1)	.70	163.7 (160.0)	412.4 (441.3)	.03
**Secondary outcomes**						
Pain intensity^d^	8.2 (2.3)	7.8 (2.5)	.40	2.9 (2.7)	5.0 (2.4)	.004
Limb circumference measurement ratio, %^e^	11.5 (7.1)	13.1 (7.6)	.40	6.6 (6.6)	17.1 (11.6)	<.001
Extent of edema in affected limb, cm^f^	35.0 (15.6)	40.1 (22.7)	.70	25.9 (15.0)	4.1 (22.7)	.002
Difference between bite site temperature and that of contralateral limb, 48 h after admission, °C^g^	2.5 (2.9)	3.1 (3.2)	.70	3.0 (5.5)	3.4 (3.6)	.22

^a^ Comparison of groups was done by the Wilcoxon rank sum test, with Bonferroni correction.

^b^ Low-intensity laser therapy combined with the regular antivenom treatment.

^c^ Regular antivenom treatment.

^d^ Pain assessment was carried out using the numerical rating scale, with values ranging from 1 to 10, 48 hours after admission.

^e^ Circumference measurement ratio (circumference of the bitten limb/circumference of the contralateral limb), 48 hours after admission.

^f^ Extent of edema in the affected limb evaluated by measuring the distance between the distal and proximal points showing swelling, 48 hours after admission.

^g^ Difference between temperature of the bite site and that of the corresponding site on the contralateral limb, 48 hours after admission.

#### Changes Over Time

**Figure 2.**
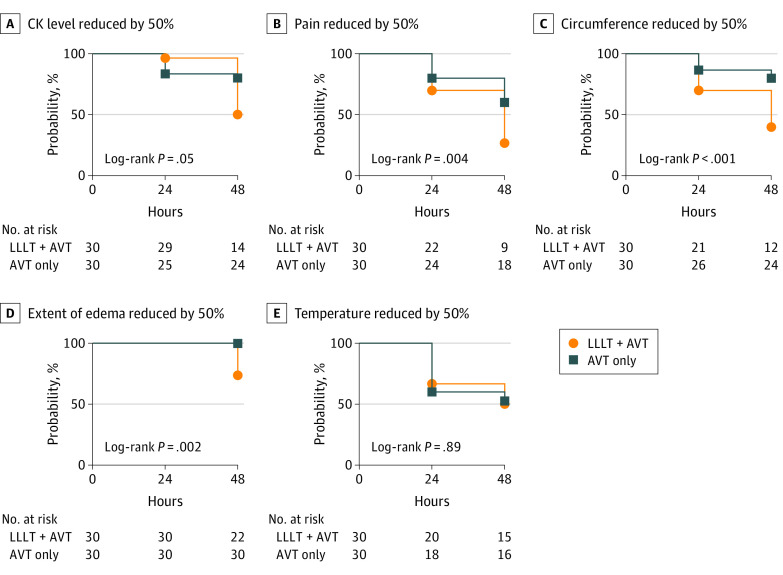
Kaplan-Meier Survival Analysis Comparing Changes Over Time Between the Low-Level Laser Therapy (LLLT) and the Comparison Group A, Time from admission to reduction of creatine kinase (CK) activity by 50%. B, Time from admission to reduction of pain intensity by 50%. C, Time from admission to reduction of circumference measurement ratio (circumference of the bitten limb/circumference of the contralateral limb) by 50%. D, Time from admission to reduction of extent of edema (distance between the distal and proximal points showing swelling) by 50%; no reduction of edema extension was observed at 24 hours in both laser and control groups. E, Time from admission to reduction of the difference between the bite site temperature and that of the contralateral limb by 50%. The log-rank test was used to test differences. The significance level of the test was *P* < .05. AVT indicates antivenom treatment.

Figure 2 shows the difference in the time elapsed from admission to the day of reduction by 50% of the evaluated assessments between the intervention and comparator groups. The time elapsed from the admission to the reduction of the creatine kinase activity by 50%, reduction in pain intensity by 50%, reduction in circumference measurement ratio by 50%, and reduction in extent of edema by 50% were significantly lower for the intervention group. Time from admission to reduction of the difference between the bite site temperature and that of the contralateral limb by 50% was similar in both groups (Figure 2).

At the 48-hour evaluation, 5 patients (17%) in the intervention group and 9 (30%) in the comparator group still required administration of metamizole (OR, 0.47; 95% CI, 0.14-1.61; *P* = .22). At the 48-hour evaluation, 1 patient (3%) in the intervention group and 3 (10.0%) in the comparator group still required tramadol (OR, 0.31; 95% CI, 0.03-3.17; *P* = .61).

The frequency of secondary infections was similar in both groups, with 27% in the intervention group and 40% in the comparator group (OR, 0.55; 95% CI, 0.18-1.62; *P* = .28). Only 3 cases of necrosis were observed, in the intervention group: 2 patients who were bitten on the finger and 1 patient who was bitten on the leg, and all of them had used a tourniquet. One of the patients who had been bitten on the finger had extensive necrosis and lost the ability to move the finger; however, they did not require amputation. No differences were found when comparing disability between the control and laser groups for each separate domain or in the summary scores (Table 3).

**Table 3.** Disability Outcomes

Disability assessment^a^	Mean (SD)		*P* value^b^
	Intervention (n = 27)^c^	Comparator (n = 27)^d^	
Cognition (domain 1)	6.7 (1.2)	6.2 (0.7)	.08
Mobility (domain 2)	6.2 (1.5)	5.7 (1.2)	.24
Self-care (domain 3)	4.1 (0.3)	4.1 (0.4)	.70
Getting along (domain 4)	5.2 (0.5)	5.2 (0.5)	.60
Life activities (domain 5)	9.2 (2.0)	8.6 (1.2)	.23
Social participation (domain 6)	8.5 (1.2)	8.9 (1.4)	.24
Summary score	39.8 (5.7)	38.8 (3.8)	.47

^a^ Disability assessment performed by phone interview from 4 to 6 months after patients’ discharge, using the World Health Organization Disability Assessment Schedule 2.0.

^b^ Comparison of groups was done by the Wilcoxon rank sum test.

^c^ Low-intensity laser therapy combined with the regular antivenom treatment.

^d^ Regular antivenom treatment.

## Discussion

The reduction of local tissue damage in *Bothrops* SBEs is a challenge to be solved with treatments that are complementary to AVT. However, the evaluation of the efficacy of therapeutic approaches for the prevention of local complications has been neglected. The information on the efficacy of anti-inflammatory drugs,^37^ PBMT,^22^ and toxin inhibitors^40^ in SBEs is restricted to the results of preclinical studies. PBMT has shown efficacy in reducing myonecrosis, inflammatory response, pain, and edema in preclinical studies^22^ but, to our knowledge, has never been tested on patients with SBE.

In recent years, the capacity of PBMT to modulate the inflammatory response, including edema and pain, as well as to enhance the healing process has led to an increase in its use in several pathological conditions. In addition, the literature shows that PBMT is a noninvasive, safe, and painless treatment.^41^ In preclinical studies, PBMT showed efficacy in reducing the local effects, such as edema, inflammatory response, pain, and myonecrosis, caused by *Bothrops* snake venoms^27,28^; however, this is the first study, to our knowledge, to examine its use in patients with SBE in a clinical study. In this study, the feasibility, safety, and efficacy of the LLLT was demonstrated in *B atrox* SBEs in a double-blind, randomized clinical feasibility trial.

A major challenge of the study was the substantial number of patients who were ineligible for recruitment, which meant that the study took longer than expected. However, the careful selection of patients ensured homogeneity between the groups.

In *Bothrops* SBEs, pain is usually the first sign to appear and alerts the patient to the situation. This manifestation is present in practically all patients.^42^ Even with AVT, pain persists for days in patients with *B atrox* envenomations and thus requires administration of analgesics.^43^ In this study, there was a significant decrease in pain intensity on the third day of follow-up in the patients who received LLLT when compared to the comparator group. Our results corroborate those of previous studies carried out in animals. Previous studies suggest that the analgesic effect of PBMT is associated with the models’^44,45^ decrease in messenger RNA levels of interleukin 6, tumor necrosis factor, and B_1_ and B_2_ kinin receptors and in the production of reactive oxygen agents that participate in the pathogenesis of *Bothrops* envenomations.^46,47^ In addition, PBMT acts by increasing the synthesis and release of endogenous opioids^48^ and directly affects pain transmission by reducing nociceptor response.^45^ In clinical trials, LLLT has proved to be effective in the control of postsurgical pain^49^ and temporomandibular disorders.^50^

Edema is also a frequent clinical manifestation in *Bothrops* envenomations.^51^ Edema varies in volume and extension and may affect only the segment of the limb where the bite occurred or, in some cases, the entire limb.^52^ In Brazil, the extent of edema is a criterion used to define the severity of the envenomation and the dosage of antivenom to be administered to the patient.^18^ In this study, edema was measured in 2 different ways, the circumference measurement ratio and the extent of the edema, both of which showed a significantly different decrease in the LLLT group over the course of the patients’ follow-up. There is evidence that LLLT improves blood flow, which increases the reabsorption of edema and the clearance of catabolites derived from the inflammatory process,^24^ as well as providing an efficient supply of nutritional and defensive elements to the injured region.^26^ This action results from the modulation of the immune system, decreases the production and release of inflammation mediators, increases the production of mitochondrial adenosine triphosphate, thus decreasing cellular oxidative stress and apoptosis, and stimulates neovascularization.^53,54,55,56^ The efficacy results of this study corroborated the efficacy observed in previous preclinical studies in animals that were experimentally subjected to *Bothrops* venom.^28,40,57^

In *Bothrops* venoms, most subtypes of phospholipase A_2_ (PLA_2_) exert myotoxic effects that may lead to necrosis and permanent tissue damage.^58^ The Lys49-PLA_2_ myotoxins induce myonecrosis via mechanisms that are independent of catalytic activity and involve the amino acids of the C-terminal region.^59,60^ Along with PLA_2_, snake venom metalloproteases have also been described as inducing myonecrosis in experimental models. The mechanism has not been fully elucidated, but previous studies have suggested that muscle damage was secondary to tissue ischemia associated with bleeding.^61^ In this study, creatine kinase levels on the third day of follow-up decreased in the intervention group, which indicates that myonecrosis was less severe in the LLLT group. Nonetheless, there was an increase in the comparator group, which indicates a worsening of local tissue injury. Our findings agree with previous reports regarding animals experimentally subjected to *Bothrops moojeni* and *Bothrops asper* venoms, in which PBMT was effective in reducing myonecrosis when observed by measuring plasma creatine kinase levels and through histological analysis.^62,63^

Technological evolution has facilitated clinical evaluation of snakebites through the use of thermography equipment that, in this study, allowed the precise delimitation of the area of inflammation for the application of LLLT, instead of using only measurement with a tape measure. In addition, the LLLT application equipment with 8 tips instead of 1 tip allowed intervention optimization with an 8-fold reduction in time taken, with concomitant application of red and infrared light, thus significantly reducing possible discomfort and the patient’s stress regarding the completion of the procedure and also contributed to minimizing possible ergonomic damage to the protocol applicator. Thus, the combination of these tools, thermography and 8-tip LLLT, benefited the evaluation and conclusion of the intervention by making it more efficient.

This study provides promising evidence for the use of LLLT in reducing the local complications of *Bothrops* envenomation, besides exerting a local analgesic effect. The results also indicated that LLLT was able to reduce myonecrosis, consequently improving the healing process in patients bitten by *Bothrops*, and thus improving their quality of life. In addition to the demonstrated efficacy, the procedure was safe.

### Limitations

This study had some limitations. This was a small, single-site clinical trial. Even though the pathophysiology and clinical characteristics of SBEs caused by different *Bothrops* species are similar, the fact that this study was conducted at a single center in the Amazon, where cases are due almost exclusively to *B atrox*, limits the generalizability of the results to other endemic sites. We suggest that larger studies be conducted to confirm our findings and to estimate the efficacy of LLLT to prevent less frequent patient-centered outcomes, especially long-term disabilities. In our study, we had resources such as thermography to limit the LLLT application area, and an 8-point laser device, which made the application faster. These resources make treatment more expensive and may limit its use in the field.

## Conclusions

In this randomized clinical trial, PBMT was feasible and was effective in reducing myonecrosis and local inflammatory effects, namely pain and edema caused by *B atrox* envenomations. This clinical trial will help to prepare the ground for a larger, more definitive trial to improve treatment for this neglected public health problem.

## Notes

Supplement 1.
Trial Protocol

Supplement 2.
eFigure 1

eFigure 2

Supplement 3.
Data Sharing Statement
