# Supplementary material for: Photobiomodulation Therapy to Treat Snakebites Caused by Bothrops atrox: A Randomized Clinical Trial
Source: JAMA Intern Med. 2023 Dec 4;184(1):70–80. doi: 10.1001/jamainternmed.2023.6538 (PMC10696517; doi:10.1001/jamainternmed.2023.6538)
Supplement: Supplement 1. — Trial Protocol [file jamainternmed-e236538-s001.pdf]

CLINICAL STUDY PROTOCOL

Efficacy of low-level laser therapy in reducing local manifestations of *Bothrops atrox* envenomations: a double-blind randomized clinical trial

VERSION NUMBER: 2.0

DATE: 6th Jun, 2018

«Any and all information presented in this document must be treated as confidential. The use of such confidential information must be restricted to the recipient for the express purpose to which it is intended and must not be disclosed, published or communicated to unauthorized persons, for any reason or in any form, without prior written consent.»

NAMES AND ADDRESSES

**STUDY MANAGER AND PRINCIPAL INVESTIGATOR**	Name	Érica da Silva Carvalho
	Address	Fundação de Medicina Tropical Dr. Heitor Vieira Dourado<br>Avenida Pedro Teixeira, 25 – Dom Pedro,<br>Manaus, Amazonas, Brasil. CEP: 69040-000.
	Telephone	+55 (92) 982822012
	E-mail	carvalhouea@gmail.com
**COORDINATOR**	Name	Jacqueline de Almeida Gonçalves Sachett
	Address	Fundação de Medicina Tropical Dr. Heitor Vieira Dourado<br>Avenida Pedro Teixeira, 25 – Dom Pedro,<br>Manaus, Amazonas, Brasil. CEP: 69040-000.
	Telephone	+55 (92) 981518086
	E-mail	jacenfermagem@hotmail.com
**MONITORING TEAM REPRESENTATIVE**	Name	Andréa Renata do Nascimento Souza
	Address	Fundação de Medicina Tropical Dr. Heitor Vieira Dourado<br>Avenida Pedro Teixeira, 25 – Dom Pedro, Manaus, Amazonas, Brasil. CEP: 69040-000.
	Telephone	+55 (92) 982151597
	E-mail	andrearenata7@hotmail.com

SUMMARY

1. SYNOPSIS 5

2. STATEMENT AS TO THE STUDY DESIGN 9

3. ABBREVIATIONS LIST 10

4. INTRODUCTION AND RATIONAL 12
4.1 Study rationale ……..…………………………………………………………………………...12
4.2 Study center ………..…………………………………………………………………………...19

5. STUDY OBJECTIVES 21
5.1 Primary …………………..……………………………………………………………………….21
5.2. Secondary ……………………………………………………………………………………..….21

6. STUDY DESIGN 22
6.1 Protocol description …………………………………………………………………………….22
6.2 Duration of the study……………………………………………………………………………22
6.3 Preliminary analysis……………………………………………………………………………..22

7. PATIENT SELECTION 22
7.1 Number of planned patients ………………..……………………………………………...22
7.2 Inclusion criteria ………………………………………………………………..……………….23
7.3 Non-inclusion criteria ………………………………………………………………………….24
7.4 Criteria for discontinuing treatment ……………………………………………………….24
7.5 Criteria for study interruption ……………………………………………………………...24
7.6 Special warnings and precautions for use …………………………………………….24

8. TREATMENTS 25
8.1 Research products ……..……………………………………………………………………..25
8.2 Method of assigning patients to the group and blinding ..……..……………………………….26
8.3 Clinical monitoring of participants ……………………………………………………………….26
8.4 Device storage conditions …..……………………………………………………………..…27
8.4.1. Environmental operating conditions ...……………...………………………………………...27
8.4.2. Environmental conditions of transport and storage...……………………………………………27
8.4.3. Device cleaning ……………...………………………………………………...…………27
8.4.4. Drums ...……………………………………………………………………………….........28
8.5 Responsibilities ………………………………………………………………………………..28

9. EVALUATION OF OUTCOMES 29
9.1 Primary …………..……………………………………………………………………….......29
9.1.1. Clinical tolerability …………………………………………………………………………29
9.1.2. Biological tolerability ...………………………………………………………………………..29
9.1.3. Assessment methods …………………………………..………………………………........30
9.2 Secondary ……………………………………………………………………………………...30

10. SAFETY 30
10.1 Safety instructions ……..……………………………………………………………………..30
10.2 Definitions of an adverse event (AE)……..………………….31

11. MANAGEMENT OF TEMPORARY OR DEFINITE DISCONTINUATION OF TREATMENT AND STUDY DISCONTINUATION 31
11.1 Permanent discontinuation of treatment............31
11.1.1. List of criteria for permanent discontinuation of treatment 32
11.2 Procedure for withdrawing patients from the study 32
11.3 Consequence 33

12. STUDY PROCEDURES 33
12.1 Scheme of visits 33
12.1.1. Inclusion visit: D1, D2, D3, D7............33
12.2 Definition of source data 35

13. STATISTICAL CONSIDERATIONS 36
13.1 Preliminary analysis............36
13.2 Statistical and analytical plans 36

14. ETHICAL AND REGULATORY STANDARDS 36
14.1 Ethical principles 36
14.2 Laws and regulations 37
14.3 Informed Consent and Assent 37

15. CONFIDENTIALITY 38

16. CLINICAL STUDY RESULTS 39

17. PUBLICATIONS AND COMMUNICATIONS 39

18. REFERENCES 40

19. APPENDICES 46

1. SYNOPSIS

**TITLE**	Efficacy of low-level laser therapy in reducing local manifestations of *Bothrops atrox* envenomations: a double-blind randomized clinical trial
**STUDY PLACE**	Fundação de Medicina Tropical Dr. Heitor Vieira Dourado
**PRINCIPAL INVESTIGATOR**	Érica da Silva Carvalho

**STUDY OBJECTIVES**	Primary:<br>To evaluate the effectiveness of low-power laser in the recovery of acute local manifestations in victims of *Bothrops* snakebites.<br>Main Secondary:<br>Evaluate the sociodemographic aspects of victims of *Bothrops* snakebites;<br><br>To describe the clinical aspects of victims of *Bothrops* snakebites with alterations in the site of the bite;<br><br>To compare different laser application protocols at sites of envenomation with inflammatory manifestations;<br><br>Evaluate the safety of the laser protocol used in the local actions of snakebite lesions.
**STUDY DESIGN**	A double-blind, 2-arm, phase II, safety, randomized clinical trial.
**STUDY POPULATION**<br>**Main selection criteria:**	Inclusion criteria<br>a) Less than 24 hours have passed since the bite;<br>b) Must be over 18 years of age;<br>c) Not having performed antivenom therapy for the current *Bothrops* snakebite at another institution.<br><br>Non-Inclusion Criteria<br>a) Patient has an established abscess or infection at the time of admission;<br>b) Patient has a dry bite;<br>c) Patient is pregnant;

	d) Patient is immunocompromised;<br>e) Patient is on a course of anti-inflammatories or antibiotics;<br>f) Patient has diabetes mellitus (DM).
**Expected total number of patients:**	60 patients, 30 in the treatment group and 30 in the control group, stratified as follows:<br><br>Arm 1) Considered the control group, this arm will receive conventional treatment from the date of admission to the hospital and will also receive the protocol with the laser turned off, therefore without any effect.<br><br>Arm 2) This arm will receive the laser treatment within 30 minutes after the infusion of antivenom serum at a dose of $4j/cm^2$.
**Expected number of centers:**	1
**STUDY PRODUCTS**	**LOW-INTENSITY LASER**: This will be a portable DMC brand, with a semiconductor light emitter (GaAIAs), the measurements used will be according to the manufacturer's protocol and the literature. Thus, this study will use as its reference the energy of 4 J, a dose of 4 J cm^2 of red and infrared laser for a time of 40 s at a power of 100 mW at 1 cm perpendicularly from the bite lesion. There will be a photographic record in all groups and stages.<br><br>**THERMOGRAPHY:** to quantify the surface temperature at the inflamed site and to measure where local blood flow will be increased or decreased. Thermographic evaluation will occur before and after each application of laser therapy in the three therapeutic moments of the intervention.

Strictly confidential

**EFFICACY CRITERIA**	• Clinical evaluation of the inflammatory aspects to identify the reduction of symptoms characterized in the process as pain, edema, redness and heat. This will be performed in all phases of the research.<br>• Laboratory tests on admission and laser applications in all groups along with assessments. The tests requested will be HMG (CBC), CK (creatine kinase), AST, ALT, LDH, CT, INR, TAP, UREA, CREATININE, VHS, SODIUM, AND POTASSIUM.
**SAFETY CRITERIA**	• Clinical tolerability: No appearance of myonecrosis.<br>• Biological safety: CK (Creatine Kinase).
**EVALUATION SCHEME**	Patients will be evaluated with the same parameters irrespective of the group to which they were allocated at the time of admission, and at 24 and 48 hours in relation to clinical and laboratory aspects.
**STATISTICAL CONSIDERATIONS**	**Number of patients:**<br>*A priori*, 60 patients in order to evaluate the effectiveness and safety of low-intensity laser in sites of snakebites involving the genus *Bothrops.*<br>**Statistical generalities:**<br>This study foresees the performance of an interim analysis when the groups reach half of their recruitment, i.e., 15 patients in each group. This analysis will be able to identify variations related to the primary outcome and will indicate the need for continuity, restructuring, or finalization of the study. Quantitative variables will be calculated for frequency and proportions, with the calculation of means.<br>Feasibility outcomes were analyzed using descriptive statistics as proportions. Trial outcomes were reported as means and standard deviations. We report the outcomes using means and standard deviations, and we conducted inferential analysis on our data in order to report the differences between the two groups. Mean values

	of creatine phosphokinase activity, pain intensity, circumference measurement ratio, extent of edema, difference between the bite-site temperature and that of the contralateral limb, and disability assessments, were compared using the t-test for independent samples, at 48 hours after admission. A Kaplan-Meier survival analysis with a log-rank test was performed to detect differences in the time elapsed from admission to the day of reduction by 50% in creatine phosphokinase activity, pain intensity, circumference measurement ratio, extent of edema and difference between the bite-site temperature and that of the contralateral limb between the intervention and comparator groups, at two points - on 24 and on 48 hours after admission. The comparison of the frequency of use of analgesics, secondary infections and necrosis in a follow-up of 48 hours, between the intervention and comparator groups, was made using the Chi-square or Fisher's exact test; odds ratios with 95% confidence intervals were obtained. The Kruskal-Wallis rank sum test was used to compare the disability scores between groups, for each domain score and for the summary score. Statistical analyses were performed in the R software in the IDE Rstudio environment (version 4.1.2), and the significance level of the tests was 0.05.
**DURATION OF THE STUDY PERIOD**	The total duration of the study will be from January 2019 to July 2021.

10.

2. STATEMENT AS TO THE STUDY DESIGN

I, Érica da Silva Carvalho, principal investigator of the study, participated in the design of this protocol and talked exhaustively about the objectives of this research and its content with the research team.

I agree to conduct the clinical trial in accordance with this protocol and to comply with its requirements, subject to ethical and safety considerations

Érica da Silva Carvalho

Principal Investigator

11.

3. LIST OF ABBREVIATIONS

WHO World Health Organization

SINAN Sistema de Informação de Agravos de Notificação

LASER Light Amplification by Stimulated Emission of Radiation

GaAs Gallium arsenide

DM Diabetes mellitus

FMT-HVD Fundação de Medicina Tropical Dr. Heitor Vieira Dourado

NNCR National Network of Clinical Research in Teaching Hospitals

MH Ministry of Health

MST Ministry of Science and Technology

SUS Sistema Único de Saúde (Unified Health System)

ABS Anti-*Bothrops* serum

ABLS Anti-*Bothrops-Lachesis* serum

ABCS Anti-*Bothrops-Crotalus* serum

GaAIAs Gallium aluminium arsenide

AIGaINP Aluminium gallium indium phosphide

HMG Hemogram

CK Creatine kinase

AST Aspartate aminotransferase

LDH Lactate dehydrogenase

**CT**	Coagulation Time
**ReBEC**	Brazilian Registry of Clinical Trials
**ICF**	Informed consent form
**UEA**	Amazonas State University
**CRF**	Clinical report form
**GCP**	Good Clinical Practice
**REC**	Research Ethics Committee

4. INTRODUCTION AND RATIONALE

4.1 Rationale of the study

Snakebites still represent a serious public health problem, especially in tropical countries due to their high frequency and high morbidity and mortality rates (1). For this reason, the World Health Organization (WHO) incorporated snakebite envenomations into its list of neglected diseases (2).

These envenomations affect, in most cases, low-income populations living in rural areas in tropical countries, thus representing a condition of poverty(3). Therefore, constant monitoring is essential to assess the extent of this important health condition and thus improve prevention and treatment measures(4). Most of the systems that control the notification of these snakebites are fragile and underestimate the real burden of the injury(5), which makes it essential to have complementary epidemiological surveillance strategies to assess the magnitude of this health problem (4).

On the South American continent, the highest number of snakebite cases is concentrated in Brazil, with between 26-29,000 cases per year, followed by Venezuela (7,000), Colombia (3,000), Ecuador (1,400-1,600), Peru (1,400-1,500) and Bolivia (1,000)(5). In Brazil, the northern region has the highest proportion of snakebite cases (520 bites/million inhabitants)(5).

In 2017, in Brazil, according to SINAN data, 28,636 snakebites were reported, of which 106 resulted in death(6). The highest incidence was observed in the northern region (49.2/100,000) with 29 deaths, followed by the northeastern region (12.6/100,000) with 40 deaths and the midwestern region (19.7/100,000) with 15 deaths. These numbers may be higher and underestimated in remote areas of the Brazilian Amazon due to considerable underreporting(6).

In the Amazon, male workers living in rural areas, especially those engaged in activities related to agriculture, hunting and forestry, are the most affected groups, strongly suggesting an occupational risk(7)(8).

A survey of indigenous communities and rubber tappers revealed that 13% of these individuals had already been bitten by snakes during their lives(7). The incidence of these envenomations by snakes correlates with the period of higher rainfall, highlighting the vulnerability of Amazonian communities, especially those living in riverine areas(9).

In these areas, among the medically important snakes involved in the envenomations, *Bothrops jararacussu*, popularly known as « jararacuçu », is found in Brazil, Bolivia, Paraguay and northeastern Argentina. In Brazil, it is also the snake with the highest venom production and injection capacity (400 mg)(10), and one of the most important venomous vipers of the viperidae family in Latin America, reaching a maximum length of 220 cm(11).

The venom of species belonging to the genus *Bothrops* has a predominantly proteolytic, coagulant, hemorrhagic, inflammatory, edematogenic and myotoxic effect(12). The clinical signs exhibited by *Bothrops* envenomations are due to the various components that are isolated from these secretions, such as the hemorrhagic factor, i.e., proteases that act in blood coagulation causing coagulation disorders, proteolytic enzymes and myotoxins(13). Among the main enzymatic proteins with myotoxic activity found in snake venoms are proteolytic enzymes and phospholipases(12)(14).

Bothrops venom can cause systemic effects, such as bleeding in pre-existing wounds, cardiovascular and renal alterations, gingival bleeding, epistaxis, hematemesis, hematuria, uterine bleeding (pregnant women), vomiting, nausea, sweating, arterial hypotension and shock(15). In envenomations by these species, the

victim will commonly have evident local effects, which may vary in intensity, and which are represented by pain and edema that sets in early and evolves progressively. Ecchymosis, bleeding, ganglionic enlargement, blisters and necrosis may also appear, and it is possible to have functional or even anatomical loss of the affected limb as sequelae(16).

Among the local reactions, cell necrosis can result in loss of tissue function in the affected limb(17). Thus, the myonecrosis caused by *Bothrops* venoms may have an indirect action as a result of the degradation of blood, vessels and ischemia caused by metalloproteinase or by the direct effect of the homologous myotoxic phospholipases A_2 (PLA_2s) on the plasmatic membrane of skeletal muscle cells(18).

Metalloproteinases, on the other hand, have a hemorrhagic action in *Bothrops* venoms, causing hemorrhage followed by myonecrosis and arterial necrosis, and enhance myotoxicity as a result of reduced muscle perfusion and circulatory deprivation, which also affects muscle regeneration(19).

An increase in the regional lymph nodes that drain the site of the bite and bruises can also be observed a few hours after envenomation, especially if there is a delay in hospital care for the patient(20). In the first 24 hours, blisters and tissue necrosis may be evident. In addition, the snakebite may present or develop complications such as bacterial infections(18), with cellulitis and/or abscess, especially in people classified as having moderate or severe injuries(6).

Thus, the inflammation triggered by envenomation plays a fundamental role in local injury, and is characterized by increased vascular permeability, formation of edema and infiltration of polymorphonuclear leukocytes and macrophages(21). However, skeletal muscle regeneration depends on the proliferation and differentiation of satellite cells, which are normally quiescent but, in response to

stimuli such as muscle trauma, become activated, proliferate and fuse with the affected fibers or with new myofibers(21).

The neutralization of this process and the clearance of the venom depends on several factors, including the potency of the antivenom, the length of time between the bite occurring and the administration of serum therapy as well as the route of administration(22). Some factors that can potentiate the local effects venom and, consequently, the appearance of complications such as the adoption of non-recommended measures, i.e., the use of constriction bands, tourniquets, incision, aspiration, and ice therapy are contraindicated(22). However, the antivenom has an immediate action on the circulating venom, but often fails to have the desired and satisfactory action at the site of the bite(23).

Several studies have invested in the search for appropriate procedures to promote muscle regeneration in cases of *Bothrops* envenomations, since antivenom has a predominantly systemic rather than local action(24). Although much progress has been made in treating the systemic effects of snake venoms, treatments for local damage are directed towards hygiene only, with a recommendation for cleaning with saline or soap and water, with cleaning as the only protocol for local care(25)(24). Within this therapeutic concept at the injury site, laboratory research in mice in controlled environments has examined the use of laser therapy as an option for tissue regeneration(25).

The laser (light amplification by stimulated emission of radiation) has started to have a prominent role in tissue repair(26). In skin lesions, the most used are the low-power ones, which generate a low-energy current in various tissues in the wound and their healing, analgesic, and anti-inflammatory effects have been observed(26). Its use began in Europe in the 60s and 70s, where significant effects were observed in the healing process. Some studies indicate and promote the use of low-intensity laser for

wound treatment and describe its benefits(27). It has been considered a valuable resource in the treatment of injuries due to its healing capacity, as it increases the proliferation of reparative cells in addition to collagen reorganization(27).

This device is made up of substances of solid, liquid, or gaseous origin that produce a beam of light, often called a “laser beam”, when excited by an energy source(28). Such a device can be classified into two categories: high-power or surgical lasers, with thermal effects showing cutting, vaporization, and hemostasis properties, and low-power or therapeutic lasers, showing analgesic, anti-inflammatory, and biostimulation properties(28). Laser therapy has photochemical, photophysical, and/or photobiological effects(29). When laser light interacts with cells and tissues at the appropriate dose, certain cellular functions can be stimulated, such as the stimulation of lymphocytes, mast cell activation and also an increase in mitochondrial ATP production, in addition to the proliferation of various cell types(28)(29)(30).

Thus, the laser has different wavelengths, the most-used, with evidence of dose-response benefit in humans, being “red” and “infrared”. The red laser has a wavelength of 660 nm and is absorbed by the mitochondria of surface cells (epithelial tissue and underlying connective tissue), increasing ATP synthesis, collagen and elastin production. In turn, the infrared laser uses a wavelength of 808 nm with absorption by the plasma membrane of the deepest cells (connective tissue, muscle, bone, cartilaginous tissue), which promotes an increase in the absorption of nutrients, water, activation of cellular metabolism and of the deep peripheral microcirculation (31).

An experiment with laser therapy applied to envenomed mice showed a significant improvement in myoglobin levels in the group irradiated with a GaAs laser from 12 hours for 7 days. Laser treatment was able to reduce local effects not only in the periods related to the degenerative phase, but mainly showed benefits for

accelerating the regeneration phase, suggesting efficacy in the treatment with low-intensity or power laser therapy(25).

The dose-response relationship was also tested in another study with mice, evaluating the intensity of 2 J/cm^2 and 4 J/cm^2 at the site of the envenomation. Laser therapy was performed for 10 consecutive days and the energy dose was deposited at the vertices of the lesion. The results showed that only the G4J showed a significant difference in revitalization(26).

The use of laser can be considered a type of care that promotes professional autonomy for its application as a procedure for prevention and treatment of people with wounds(27). Its use is an important adjuvant in the healing process of chronic lesions, evidenced by the reduction in the dimensions of the lesion and its re-epithelialization(28). Since it has a quick effect, it has been shown to promote increased vascularization, with the appearance of granulation tissue, and faster healing, accelerating the process of cell proliferation(29).

There are some protocols for the application of laser therapy in acute and chronic lesions, such as in the oral mucosa(30). Low-intensity laser has been used to treat mouth ulcers, speed up tissue repair, and reduce edema and pain, since phototherapy demonstrates anti-inflammatory action(25)(30). Endre Mester, in 1967, in Hungary, was one of the first researchers to use low-intensity laser to show its effects on difficult-to-heal ulcers in humans, and obtained approximately 90.2% cure, 16.4% improvement, and 8.7% non-response(32). Therefore, the low-intensity laser has a positive effect on the acceleration of these lesions, as well as on the reduction of symptoms(32).

The quality of life of patients with oral mucositis induced by antineoplastic treatments was evaluated after the experimental application of laser therapy and also

after the regression of oral lesions(33). This experimental trial with 18 cancer patients treated for oral mucositis used a Quality of Life Questionnaire (UW-QOL) applied before the sessions with a low power laser and after the regression of the lesions(33). Quality of life improved after laser therapy sessions, with the most significant changes occurring in domains related to pain, appearance, swallowing, chewing, speech, taste and salivation, with the low-power laser being proved to be an adequate tool in the management of oral mucositis(33).

Another clinical trial with the use of lasers in the treatment of aggressive periodontitis was compared to conventional mechanical treatment associated with drug therapy and oral hygiene instruction, in which 15 patients were randomly divided into four groups (G1: mechanical therapy, G2: mechanical therapy + 1 application of laser therapy, G3: mechanical therapy + 2 applications of laser therapy and G4: mechanical therapy + 3 sessions on days 0, 7 and 21)(34).

In all groups, there was a statistical reduction in the 25 clinical and microbial parameters evaluated, but group 4, which used three sessions of laser therapy, showed a better reduction than the other groups(34). According to the authors, photodynamic therapy would be a valuable complementary tool in the treatment of aggressive periodontitis(34).

Foot ulcers in severe complications of diabetes mellitus were also evaluated for the effectiveness of low-power laser therapy in the healing dynamics of these ulcers(35). This was a trial involving 68 patients with type 2 DM and grade I Meggitt-Wagner ulcers of at least 4 weeks' duration with an intervention group and a control group. Healing or percentage reduction in the ulcer area during a period of 15 days after starting treatment was recorded(35). The percentage of ulcer area reduction was 40.24 ± 6.30 mm^2 in the study group and 11.87 ± 4.28 mm^2 in the control group ($p<0.001$, Z0.08.08). Laser therapy is therefore beneficial as an adjunct to conventional therapy in the treatment of diabetic foot ulcers(35). The regeneration of injured tissue in

diabetes mellitus with the use of lasers also demonstrates economic and social benefits and a decrease in public health expenditures, since the cost of materials for treating and cleaning ulcers is increasing(36).

In studies in mice with snakebite injuries, doses of 4 J/cm² show successful results in terms of faster healing speeds and pain reduction(25,26,28).

The effects of low-intensity laser in accelerating wound healing have been attributed to the stimulation of various biological systems, such as increased cell proliferation and activity, increased DNA synthesis, modulation of growth factor production, and reduced prostaglandin production(37).

Faced with the high prevalence of disabling necrotic lesions secondary to snakebites in the Amazon region, complications in injuries can cause abstinence from work activities and decrease income generation. the benefits of low-intensity laser therapy in tissue repair and the reduction of pain symptoms makes it is necessary to investigate the effects of this type of therapy in snakebite injuries. This, therefore, raises the need for further investigations using low-power lasers in snakebite wounds in patients seeking care in the public health system, to establish clinical and preventive protocols laser use in snakebite wounds in humans as we have already found good results in rats.

This project proposes the use of low-intensity laser on the skin in places where *Bothrops* venom is present in order to minimize local damage and reduce inflammatory effects, thus leading to faster recovery and prevention of complications such as local necrosis. Since the literature has demonstrated safety and efficacy in the application of laser therapy in mice without causing harm, this makes testing in humans necessary, and in the current scenario there is nothing for the local treatment of envenomations.

a. 4.2 Study Center

The Fundação de Medicina Tropical Heitor Vieira Dourado (FMT-HVD) is located in the city of Manaus, Amazonas, and is considered a national and world reference center for the treatment of tropical diseases, especially for events related to venomous animals. currently, it represents the reference center for infectious, parasitic, and dermatological diseases in the state of Amazonas, with 40 medical offices and more than 250,000 health consultations/year; 150 beds, 10 of which are in the intensive care unit; 14 beds dedicated to clinical research; an electronic medical record for medical information with Idoctor® system, allowing faster monitoring in clinical trials; a clinical analysis laboratory and staff trained in good clinical practices).

In addition, since 2005, the foundation has been part of the National Network of Clinical Research in Teaching Hospitals (NNCR), which is an initiative of the Ministry of Health (MH) and the Ministry of Science and Technology (MST) that seeks to promote best research practices geared towards the needs of the public health network.

The network prioritizes the development of clinical trials of drugs, procedures, equipment, and diagnostic devices, of interest to the SUS. The objective of these 19 centers is that the regional training resulting from targeted national integration promotes the acceleration of the growth and scientific capacity of each of these centers. In this way, the centers will be able to develop their own tools capable of solving country-specific challenges.

In this sense, individuals treated in the hospital network in the state of Amazonas are referred to the service in question for the treatment and follow-up

of those involved in snakebites, as this unit is a reference for this type of health problem in the state. In Manaus, FMT-HVD is the only hospital unit that dispenses snakebite antivenom.

The snakebite victims hospitalized at FMT-HVD in the period from 1974 to 1984 registered a total of 514 victims. From 1974 to 2012, with an increase in the incidence of snakebites in the 1990s, which peaked in 1998, with 554 cases at FMT-HVD, since the beginning, the epidemiological surveillance of snakebites in the state of Amazonas has shown that rural activities are closely associated with the occurrence of this problem. In the 1990s, the development of sustainable forestry, fishing, and agriculture in the state was stimulated as a result of official government support for the creation of the free green zone program.

Thus, FMT-HVD represents an important site for the development of this clinical study in the face of a sufficient number of attendances for the recruitment of victims of *Bothrops* envenomations.

5. STUDY OBJECTIVES

5.1 Primary

To evaluate the effectiveness of low-power laser in the recovery of acute local manifestations in victims of *Bothrops* envenomations.

Clinical and biological tolerability:

- Clinical tolerability: No appearance of myonecrosis.
- Biological safety: CK (creatine kinase).

5.2. Secondary

Evaluate the sociodemographic aspects of victims of *Bothrops* envenomations;

Describe the clinical aspects of victims of *Bothrops* envenomations with alterations in the site of the bite;

Compare different laser application protocols at sites of envenomation with inflammatory manifestations;

Evaluate the safety of the laser protocol in regards to the local effects of snakebites.

6. STUDY DESIGN

6.1. Protocol description

A 2-arm, double-blind, randomized, safety, phase II clinical trial conducted at one center in Brazil.

b. 6.2 Study duration

All patients presenting with FMT-HVD bitten by a *Bothrops* snake will be submitted to the standard evaluation. Selected participants will be randomized to one of three treatment regimens at the time. The treatment period will last for 3 days, with 3 days of laser application. The study will run from January 2019 to July 2021.

After the follow-up of the study, the patient will be instructed to seek the study center at any time if they have a complaint related to the snakebite.

6.3 Preliminary analysis

A preliminary evaluation is planned for this study after the inclusion of the first 46 patients, 23 in each arm, to validate the observed proportion of improvement in the local inflammatory condition in patients and, finally, recalculate the number of patients to be included.

7. PATIENT SELECTION

7.1. Number of patients planned

The research patients will be allocated into 2 main arms:

- **Arm 1:** Thirty patients bitten by *Bothrops* snakes who received regular FMT treatment.

- **Arm 2:** These patients will receive the laser within 30 minutes after administration of the antivenom serum at a dose of 4 J/cm^2.

First phase T1 - first laser application and first clinical evaluation will take place as follows:

- Arm 1, considered control, will receive the conventional treatment from the date of admission to the hospital and will also receive the laser, but turned off, therefore without any effect.

- Arm 2, will receive the laser within 30 minutes after administration of the antivenom serum at a dose of 4 J/cm^2.

Second phase T2 - The groups will receive the same laser therapy regimen to which they were randomized within 24 hours.

Third phase T3 - 48 hours after the first application. On the 3rd day, the patient is discharged from the hospital if they are well.

7.2. Inclusion criteria

Patients eligible for the study must meet the following characteristics:

a) Less than 24 hours have passed since the bite;

b) Must be over 18 years of age;

c) Not having performed serum therapy for the current *Bothrops* snakebite at another institution.

7.3. Non-inclusion criteria

a) Patient has an established abscess or infection at the time of admission;

b) Patient has a dry bite:

c) Patient is pregnant;

d) Patient is immunocompromised;

e) Patient is on a course of anti-inflammatory or antibiotic drugs;

f) Patient has diabetes mellitus (DM).

No waiver, prospective or retrospective, of deviation in any way from the inclusion/exclusion criteria can be granted to the investigators.

7.4. Criteria for discontinuing treatment

Treatment can be stopped if there is an increase in CK or inflammatory markers: pain, heat, edema, and redness or in cases of secondary infection.

7.5. Criteria for study interruption

If the study does not show benefits, for example, the control group shows improvement and the low-power laser groups do not. The presence of marked myonecrosis will also indicate the need for the discontinuation of the study.

7.6. Special warnings and precautions for use – contra-indications

The laser must not be used for:

- Eye irradiation;
- Irradiation of the fetus or uterus in pregnant patients;
- Irradiation of bleeding areas;
- Irradiation of infected areas;
- Irradiation of an area with heat hypoesthesia;
- Irradiation of the epiphyseal lines in children;
- Irradiation in children younger than 2 years old;
- Irradiation of thyroid gland, endocrine glands and testes;
- Irradiation of vagus nerves;

- Irradiation in areas with malignant tumors or cancer;
- Irradiation of the area in patients who are receiving dermatological treatment and are using photosensitive substances or in patients who are using drugs or substances that may have a photosensitizing action.

8 TREATMENTS

8.1. Research products

The action of the laser is therapeutic and for its clinical application, the dosimetry comprises the following aspects:

A) Energy = active principle of phototherapy represented in joules (J)

B) Dose = amount of energy per unit transferred to the tissue, measured in J/cm^2.

C) Wavelength (CO) = defines the actions of the red laser (most superficial layer) known in the device as L1 of 660 nm of CO and infrared that reaches deeper tissues, in the device it is seen as L2 and has a wavelength of 808 nm.

D) Light beam power is the same for both red and infrared types, always at 100 mW.

The low-intensity laser will be the portable DMC brand (Figure 1), with the semiconductor light emitting (GaAIAs), the measures used will be those of the manufacturer’s protocol and will also be based on studies of envenomation in mice(25,26, 28). Thus, this study will use as a reference the energy of 4 J, a dose of 4 J/cm^2 in red and infrared laser for a period of 40 s with the power of 100 mW at 1 cm perpendicularly away from the bite lesion. There will be a photographic record in all groups and stages.

According to Ortiz (2001), the biomodulation effects that occur with emissions greater than 10 J per cm^2 can cause phagocytosis of leukocytes. The measure of inhibition of phagocytosis is up to 4 J cm^2. The effect of phagocytosis is avoided if the correct laser dose is applied and the device is calibrated every 6 months.

Thermography will be performed to quantify the surface temperature at the inflamed site and to measure where local blood flow is increased or decreased. Thermographic evaluation will occur before and after each application of laser therapy in the three therapeutic moments of the intervention.

8.2. Method of assigning patients to the group and blinding

A randomized clinical trial is a prospective study in humans comparing the effect and value of an intervention against a control.

Randomized clinical trials should be considered when:

A. There is uncertainty about the effect of an exposure or treatment;

B. Exposure can be modified in the study.

Potential limitations of randomized controlled trials include:

A. Limited generalizability of the study population.

B. Limited generalization of the environment under study.

C. Randomized clinical trials answer a specific study question.

The usual measures of the magnitude of effect in randomized clinical trials are relative risk and risk difference.

The method that will be used in the measurement of the research is double masking (double-blind). This implies that neither the patient nor the professional doing the evaluation knows which treatment is being given. The double-blind method tries to eliminate evaluation bias.

8.3. Clinical monitoring of participants

During this period, clinical (1x/day) and laboratory (1x/day) examinations will be performed daily. Considering the national recommendation, this study will classify the patient according to the local aspects of the lesion for laser application:

Local Aspects	Edema	Pain (numerical scale)	Temperature (contralateral difference)
Light	1 to 2 segments	0-3	Up to 0.5 ºC
Moderate	3 to 4 segments	4-7	0.6-1 ºC
Severe	5 segments	7-10	More than 1 ºC

* ADAPTED (38).

c. Participants in arms 1 and 2 will receive clinical evaluation and classification and tests related to inflammation and myonecrosis will be performed during the 3 days of hospitalization.

d.For the evaluation, a clinical form will be used with data on local and systemic signs and symptoms, daily evolution of the patient, evaluation of the wound site at all verification times, demographic data and laboratory tests, and laser application. (see Appendix A).

e. 8.4. Device storage conditions

8.4.1 Environmental operating conditions

- Room temperature +15 °C to +30 °C
- Relative humidity 30% to 75% (non-condensing)
- Atmospheric pressure 700 hPa to 1,060 hPa

8.4.2 Environmental transport and storage conditions

- Room temperature -12 °C to +50 °C
- Relative humidity 0% to 90% (non-condensing)
- Atmospheric pressure 700 hPa to 1,060 hPa

8.4.3 Device cleaning

- For cleaning, bactericidal substances, such as 70% alcohol or surface disinfectant, can use be used. Do not let liquid penetrate inside the case or battery charger;
- Glasses can be cleaned by washing with water and neutral soap, drying lightly with paper tissues;
- The nozzle of the laser pen has a micro lens and a glass window at the beam output. This nozzle has metal and glass parts; therefore, it can be autoclaved and, periodically, the external parts of the window and micro lens must be cleaned with cotton or tissue paper (slightly moistened with alcohol) to remove residues that alter the output power of the beam.
- The pen body cannot be immersed in liquids (water, alcohol, solvent, etc.);
- The pen cannot be placed in ovens or autoclaves. Only the nozzle can be placed in autoclaves;
- Keep the battery contact terminals clean at all times. Use only a clean, dry cloth to clean the terminals.

8.4.4 Battery

- Do not expose the battery to high temperatures (above +45 °C in operation or load) or fire due to risk of explosion;
- Always keep the battery charged, always leave the battery charging when not in use;

- Charge the battery in an environment that is at room temperature;
- Use only the charger that comes with the equipment;
- Do not use the flexible battery charger power cable if parts of it are squashed or if the insulation is damaged;
- Do not short-circuit the battery terminals;
- Do not allow metallic objects to make contact with the battery terminals;
- If the equipment remains stored for a long period, recharge the battery every 6 months;
- The battery must only be replaced by authorized technicians.

f. 8.5 Responsibilities

The investigator or other staff member permitted to store the laser is responsible for ensuring that the product used in the clinical trial is maintained safely as specified by the manufacturer and in compliance with applicable regulatory requirements.

All products must be distributed according to the study arm, by a previously designated researcher to ensure that an accurate record is maintained. A record of all study evaluations will be maintained in the investigator's file at the site (ISF) for the study. Data regarding the date of receipt, energy level, number of points, applications and date of use will be recorded for each laser application. A laser logbook will be maintained at the study clinic.

9. EVALUATION OF OUTCOMES

9.1. Primary

g. Decreased inflammation.

9.1.1 Clinical tolerability

i. Clinical data will be collected from the site of the bite and its evolution in the first 3 days, observing all and any signs. At each visit, the investigator will ask the patient about their symptoms, whether they have had any adverse events since their last visit, in addition to performing a standard physical examination.

ii. 9.1.2 Biological tolerability

Assessments of laboratory exams will be performed on admission and laser applications in all groups along with the assessments. The tests requested will be HMG (CBC), CK (creatine kinase), AST (aspartate aminotransferase), LDH (lactic dehydrogenase), and CT (coagulation time).

Blood samples for hematology and clinical biochemistry will be collected from patients via venipuncture. About 10 ml of blood will be collected from each patient for hematology during these visits.

- Hematological parameters: erythrocytes, hemoglobin, platelets, leukocytes and reticulocytes. These parameters will be obtained from a complete blood count and CT.

Strictly confidential

- Biochemical parameters: blood levels of creatinine, AST, LDH. These parameters will be measured according to the enzymatic assay methods for each parameter.

9.1.3 Assessment methods

Physical and clinical signs and symptoms: the following local signs and symptoms will be systematically examined by the investigator and described as being absent or present, with grading of intensity depending on the sign/symptom: pain, redness, edema and heat (temperature).

9.2 Secondary

Prevention of myonecrosis

Efficacy should be evaluated according to the results of creatine kinase (CK) in the follow-up period (see Appendix A).

• The increase in CK indicates myonecrosis, which should be investigated from the first moment with the patient and after 24 h, 48 h and 72 h. When the patient returns on the 7th day, CK testing will also be performed and evaluated in the two arms of the clinical trial.

Other secondary parameters include:

• Proportion of any adverse events, such as itching, skin redness, changes in skin pigmentation, bruising, scarring, peeling, skin depression, and textural and thickness changes.

12. SAFETY

10.1. Safety instructions

a. The safety and tolerability of the treatment will be evaluated based on the registration of adverse events (AEs) and the classification of laboratory evaluations and vital signs. A severity rating scale, based on the local symptomatology rating scales of all symptoms, physical examination findings, and test results will be used (see Appendix B and C).

b. All laboratory abnormalities (see Appendix C)

10.2 Definitions of an adverse event (AE)

An adverse event is any undesired medical occurrence in a patient or clinical trial participant who is administered a pharmaceutical product; such an occurrence need not necessarily be causally related to the treatment.

A priori, the safety outcomes specified in the protocol will not be considered as AEs unless, due to the evolution, severity or any other characteristics of these events that exceed the established safety limits, the investigator, according to their clinical judgment.

In this study, adverse events, such as itching, skin redness, changes in skin pigmentation, bruising, scarring, peeling, skin depression, and textural and thickness changes, will be described in both arms, during the follow-up.

11. MANAGEMENT OF TEMPORARY OR DEFINITE DISCONTINUATION OF TREATMENT AND STUDY DISCONTINUATION

Any discontinuation of the experimental product must be fully recorded on the CRF. In any case, the patient must remain in the study for the period of 3 days of hospitalization and return if there is any discomfort or worsening of the clinical condition.

11.1 Permanent discontinuation of treatment

c. Permanent discontinuation of treatment means any discontinuation associated with the definitive decision by the investigator or the patient not to re-expose the patient to the research product.

11.1.1 List of criteria for permanent discontinuation of treatment

Patients can withdraw from the treatment of their own free will, at any time and regardless of reason, or this can also be decided by the investigator. Every effort should be made to document the reasons for discontinuing treatment in the CRF.

The objective criteria for discontinuing the study treatment and adopting a safe treatment are as follows:

- Revocation of the informed consent form: The patient or his parents or guardians may withdraw from treatment with the Experimental Product if they so decide, at any time and regardless of justification, or this may be by decision of the Investigator,
- Onset of infection.
- Manifestation of an adverse event that justifies discontinuation of treatment.

11.2 Procedure for withdrawing patients from the study

Patients may withdraw from the study, before its end, if they so wish, at any time and regardless of justification, or this may occur by decision of the investigator.

Enrolled patients will be withdrawn from the study for the following reasons:

1. Revocation of the informed consent form
2. Development of any serious adverse event requiring study interruption
3. Inability to comply with the study design and procedures
4. Diagnosis of a serious chronic illness that requires frequent medical care.

11.3 Consequence

Patients who are withdrawn from the study cannot be re-enrolled. Your inclusion number and treatment number must not be used again. In specific situations to be discussed between the investigator and sponsor, patients who have not yet been randomized may be re-included in the study.

12 STUDY PROCEDURES

12.1 Scheme of visits

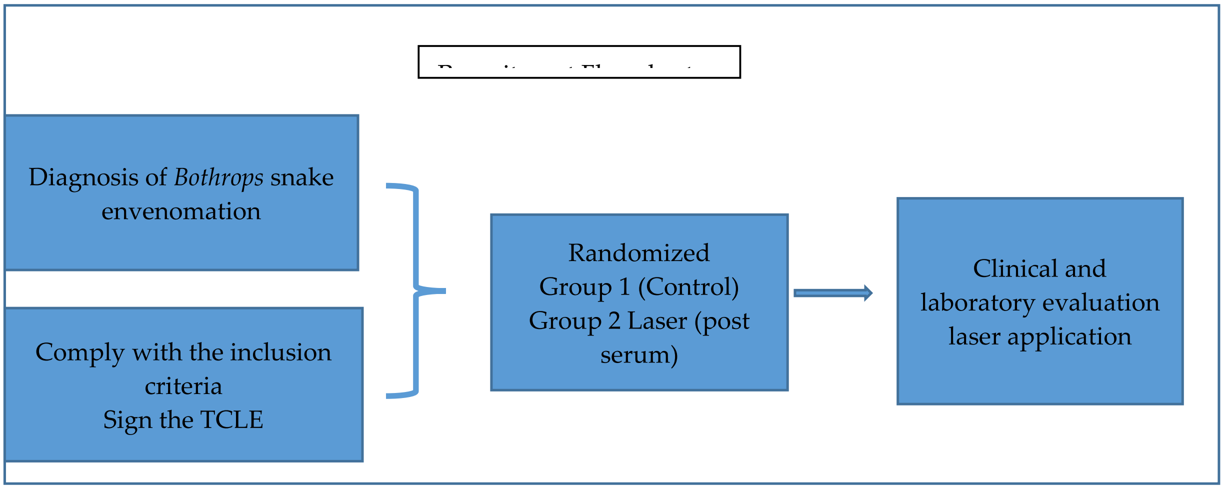

Figure 1 - Recruitment Flowchart

12.1.1 Inclusion visit: D1, D2, D3

On the first day **(D1),** the patient is seen and randomized to one of the 2 groups.

- In Arm 1, patients will be part of the control, where after completing the clinical form, the signs and symptoms and collection of laboratory tests, they will receive the application of the low-power 4J laser at the site of the snakebite, but turned off.

- In Arm 2, patients, after completing the clinical form, the signs and symptoms and collection of laboratory tests, will receive the application of a low-power 4J laser at the site of the snakebite 30 minutes after the infusion of Antivenom (AV).

On the second day **(D2),** 24 hours after the first application, patients were randomized:

- In Arm 1, the control patients, after verifying the signs, symptoms and collection of laboratory tests, will receive the application of a low-power 4J laser at the site of the snakebite, but turned off.

- In Arm 2, patients will receive after the signs, symptoms and collection of laboratory tests, will receive the application of low- power laser in 4J at the site of the bite.

On the third day **(D3),** 48 hours after the first application, patients were randomized:

- In Arm 1, the control patients, after verifying the signs, symptoms and collection of laboratory tests, will receive the application of a low-power 4 J laser at the site of the snakebite, but turned off.
- In Arm 2, patients, after verifying the signs, symptoms and collection of laboratory tests, will receive the application of a low-power 4J laser at the site of the bite.

12.2 Definition of source data

All data and information requested in the clinical record (CRF) will be collected in a patient file (iDoctor medical record) after interviewing the patient, including vital and clinical signs, laboratory tests, concomitant illnesses and any possible concomitant medications

Dates of visits and demographic data will be recorded on the CRF along with printed laboratory test results and will be filed with the patient's medical file. Treatments will be recorded in a file or spreadsheet created for this purpose.

13. STATISTICAL CONSIDERATIONS

13.1 Preliminary analysis

After the admission of the first 30 patients, this study foresees a preliminary analysis for the validation of the application of the low-power laser in arms 1 and 2, to eventually recalculate the number of patients to be included or not.

13.2 Statistical and analytical plans

Feasibility outcomes were analyzed using descriptive statistics as proportions. Trial outcomes were reported as means and standard deviations. We report the outcomes using means and standard deviations, and we conducted inferential analysis on our data in order to report the differences between the two groups. Mean values of creatine phosphokinase activity, pain intensity, circumference measurement ratio, extent of edema, difference between the bite-site temperature and that of the contralateral limb, and disability assessments, were compared using the t-test for independent samples, at 48 hours after admission. A Kaplan-Meier survival analysis with a log-rank test was performed to detect differences in the time elapsed from admission to the day of reduction by 50% in creatine phosphokinase activity, pain intensity, circumference measurement ratio, extent of edema and difference between the bite-site temperature and that of the contralateral limb between the intervention and comparator groups, at two points - on 24 and on 48 hours after admission. The comparison of the frequency of use of analgesics, secondary infections and necrosis in a follow-up of 48 hours, between the intervention and comparator groups, was made using the Chi-square or Fisher's exact test; odds ratios with 95% confidence intervals were obtained. The Kruskal-Wallis rank sum test was used to compare the disability scores between groups, for each domain score and for the summary score. Statistical analyses were performed in the R software in the IDE Rstudio environment (version 4.1.2), and the significance level of the tests was 0.05.

14. ETHICAL AND REGULATORY STANDARDS

14.1 Ethical principles

This clinical study will be carried out following the Resolutions 466/2012 and 580/2018, and the relevant Brazilian regulations.

The study will be forwarded through the Platforma Brasil to the Research Ethics Committee of the Amazonas State University and will be registered in the Brazilian Registry of Clinical Trials (ReBEC). Patients must sign the ICF to participate in the research.

This clinical trial was registered on a website (Registro Brasileiro de Ensaios Clínicos - ID RBR-2rcm2g) before the inclusion of the first patient. It presents basic information about the trial that is sufficient for interested patients (and healthcare professionals) to know how to participate in the trial.

This study was registered in the Brazilian Registry of Clinical Trials (ReBec): RBR-4qw4vf and UTN Number: U1111-1244-4898.

14.2 Laws and regulations

This clinical trial will be carried out in compliance with all international laws and regulations and with the laws and regulations of Brazil, where the clinical trial is carried out.

14.3 Informed consent and assent

The investigator (in accordance with applicable regulatory requirements), or someone designated by the investigator, but under the investigator's

responsibility, shall inform the patient or the patient's parents or guardians of all the pertinent aspects of the clinical trial, including from written information outlining the approval/favorable opinion from the local REC. All participants should receive as much information about the study as possible, in language and terms they can understand.

Before a patient participates in the clinical trial, the written informed consent form and any other applicable local documents in accordance with local laws and regulations must be signed, with the name and date filled in by the patient or their legal representative, and by the person who talked about the informed consent form. The patient should keep a copy of the signed and dated informed consent form.

- Participants who can read the informed consent form/assent form must do so before entering their name and date and signing the form.

- In the case of participants who know how to write, but not how to read, the informed consent form/assent form will be read aloud before they enter their name and date.

- In the case of participants who can understand, but who cannot read and write, the informed consent form will be read aloud in the presence of an impartial witness, who will sign and date the informed consent form to confirm that consent has been given.

The informed consent form used by the investigator to obtain the patient's informed consent must be evaluated and approved by the sponsor prior to submission to the relevant Ethics Committee for approval/favorable opinion.

15. CONFIDENTIALITY

All information disclosed or provided by patients or produced during the clinical trial, including, but not limited to, the clinical trial protocol, the CRFs, the investigator's brochure and the results obtained during the trial are confidential.

The investigator and anyone under the investigator's authority agrees to keep this information confidential and not to disclose this information to any third party.

However, the presentation of this clinical trial protocol and other necessary documents to the ethics committee is expressly permitted, bearing in mind that members of ethics committees have the same duty of secrecy.

The investigator and sub-investigators must use the information exclusively for the purposes of the clinical study, avoiding any personal use or in favor of a third party.

16. CLINICAL STUDY RESULTS

The researcher will be responsible for preparing a clinical study report. Regardless of the outcome of the study, the researcher undertakes to publish the results.

17. PUBLICATIONS AND COMMUNICATIONS

The researcher recognizes the right to use data from the clinical study in courses, conferences and scientific publications. However, to ensure the accuracy and scientific value of the information, while preserving the investigator's independence and responsibility, and the confidentiality of the information, only

verified and validated data will be used. For this, it is essential that the parties agree before any publication or communication is carried out by the investigator.

Study results must be submitted for review by the steering committee prior to publication. All study investigators and committee members fully authorize the steering committee to present or publish priority results. However, in the absence of publication within 12 months of completion of the clinical trial at all centers, the steering committee, if applicable, may consider the investigator's request for independent publication. If no publication occurs within 12 months of the end of the clinical trial, the investigator shall have the right to independently publish the results of that clinical trial, subject to the review procedure set forth herein. The steering committee may stipulate specific rules for publication.

No further publication is permitted prior to primary publication. Any presentations or subsequent publications made by study participants must be approved by the researcher in charge, making reference to the study and the primary publication.

18. REFERENCES

1. De Sousa EA, Bittencourt JAHM, De Oliveira NKS, Henriques SVC, Picanço LCDS, Lobato CP, et al. Influence of a low-level semiconductor gallium arsenate laser in experimental envenomation induced by *Bothrops atrox* snake venom. Am J Pharmacol Toxicol. 2012;7(4):141–8.

2. Feitosa EL, Sampaio VS, Salinas JL, Queiroz AM, Da Silva IM, Gomes AA, et al. Older age and time to medical assistance are associated with severity and mortality of snakebites in the Brazilian Amazon: A case-control study. PLoS One. 2015;10(7):1–15.

3. Gutiérrez JM, Theakston RDG, Warrell DA. Confronting the neglected problem of snake bite envenoming: The need for a global partnership. PLoS Med. 2006;3(6):0727–31.

4. Chippaux JP. Snake-bites: Appraisal of the global situation. Bull World Health Organ. 1998;76(5):515–24.

5. Cupo P. Bites and stings from venomous animals: A neglected Brazilian tropical disease. Rev Soc Bras Med Trop. 2015;48(6):639–41.

6. Ministério da Saúde. ACIDENTE POR ANIMAIS PEÇONHENTOS [Internet]. Sistema de Informação de Agravos de Notificação - SINAN. 2017. Available from: http://portalsinan.saude.gov.br/

7. Pierini S V., Warrell DA, De Paulo A, Theakston RDG. High incidence of bites and stings by snakes and other animals among rubber tappers and Amazonian Indians of the Jurua valley, acre state, Brazil. Toxicon. 1996;34(2):225–36.

8. Moreno E, Queiroz-Andrade M, Lira-da-Silva RM, Tavares-Neto J. Clinical and epidemiological characteristics of snakebites in Rio Branco, Acre. Rev Soc Bras Med Trop. 2005;38(1):15–21.

9. Nascimento SP Do. Aspectos epidemiológicos dos acidentes ofídicos ocorridos no Estado de Roraima, Brasil, entre 1992 e 1998. Cad Saude Publica. 2000;16(1):271–6.

10. Correa-Netto C, Teixeira-Araujo R, Aguiar AS, Melgarejo AR, De-Simone SG, Soares MR, et al. Immunome and venome of *Bothrops jararacussu:* A proteomic approach to study the molecular immunology of snake toxins. Toxicon. 2010;55(7):1222–35.

11. Milani Júnior R, Jorge MT, de Campos FP, Martins FP, Bousso a, Cardoso JL, et al. Snake bites by the jararacuçu (*Bothrops jararacussu*): clinicopathological studies of 29 proven cases in São Paulo State, Brazil. QJM. 1997;90(5):323– 34.

12. F.M.O. Pinho IDP. Ofidismo. Rev Ass Med Bras. 2001;47(1).

13. Cunha E, Martins OA. Principais Compostos Químicos Presente Nos Venenos De Cobras Dos Gêneros *Bothrops* e *Crotalus* – Uma Revisão. Rev Eletrônica Educ e Ciência. 2012;02(2):21–6.

14. Tashima AK, Zelanis A, Kitano ES, Ianzer D, Melo RL, Rioli V, et al. Peptidomics of Three *Bothrops* Snake Venoms: Insights Into the Molecular Diversification of Proteomes and Peptidomes. Mol Cell Proteomics [Internet]. 2012;11(11):1245–62. Available from: http://www.mcponline.org/lookup/doi/10.1074/mcp.M112.019331

15. Ministério da saúde do Brasil. Acidentes por Lepidópteros. Manual de diagnóstico e tratamento de acidentes por animais peçonhentos. 2001. 120 p.

16. Santos CMA, Carvalho, Carolina Novaes, Figueiredo GA de M.

Sistema de Informação de Agravos de Notifi cação–Sinan: normas e rotinas. 2006. 80 p.

17. Barbosa AM, Villaverde AB, Sousa LG, Munin E, Fernandez CM, Cogo JC, et al. Effect of low-level laser therapy in the myonecrosis induced by *Bothrops jararacussu* snake venom. Photomed Laser Surg. 2009;27(4):591–7.

18. Sobrinho JC, Kayano AM, Simões-Silva R, Alfonso JJ, Gomez AF, Gomez MCV, et al. Anti-platelet aggregation activity of two novel acidic Asp49- phospholipases A2from *Bothrops brazili* snake venom. Int J Biol Macromol [Internet]. Elsevier B.V.; 2018;107(PartA):1014–22. Available from: https://doi.org/10.1016/j.ijbiomac.2017.09.069

19. Queiroz LS, Santo Neto H, Assakura MT, Reichl AP, Mandelbaum FR. Pathological changes in muscle caused by haemorrhagic and proteolytic factors from *Bothrops jararaca* snake venom. Toxicon. 1985;23(2):341–5.

20. Dourado DM, Fávero S, Baranauskas V, Da Cruz-Höfling MA. Effects of the Ga-As Laser Irradiation on Myonecrosis Caused by *Bothrops Moojeni* Snake Venom. Lasers Surg Med. 2003;33(5):352–7.

21. Vasculares A, Bechara GH, Szabó MPJ. PROCESSO INFLAMATÓRIO. 1. Alterações Vasculares e Mediação Química. Medicina (B Aires). 1926;1–15.

22. Sachett JAG, da Silva IM, Alves EC, Oliveira SS, Sampaio VS, do

Vale FF, et al. Poor efficacy of preemptive amoxicillin clavulanate for preventing secondary infection from *Bothrops* snakebites in the Brazilian Amazon: A randomized controlled clinical trial. PLoS Negl Trop Dis. 2017;11(7):1–21.

23. Jorge MT, Ribeiro L a. Dose de soro (antiveneno) no tratamento do envenenamento por serpentes peçonhentas do gênero *Bothrops*. Rev Assoc Med Bras. 1997;43(1):74–6.

24. Tokarnia CH, Peixoto PV. A importância dos acidentes ofidicos como causa de mortes em bovinos no Brasil. Pesqui Vet Bras. 2006;26(2):55–68.

25. Dourado DM, Matias R, Almeida MF, De Paula KF, Vieira RP, Oliveira LVF, et al. THE EFFECTS OF LOW-LEVEL LASER ON MUSCLE DAMAGE CAUSED BY *Bothrops neuwiedi* VENOM. J Venom Anim Toxins incl Trop Dis. 2008;14(3):423–34.

26. Silva TS da, Mendes F, Alves ÂMP, Alves ÉPB, Bertolini GRF. Estudo microscópio da lesão tecidual em pele de ratos Wistar, tratados com laser de baixa potência. Rev Bras Biociências. 2010;8(3):264–7.

27. Ferreira A, Candido M, Candido M. O Cuidado de Pacientes com Feridas e a Construção da Autonomia do Enfermeiro. Rev Enferm UERJ. 2010;18(4):656– 60.

28. Souza DM De. Laserterapia No Tratamento De Ferida Aberta Crônica Pós - Queimadura : Um Estudo De Caso Laser Therapy in the Treatment of Chronic.

Strictly confidential

:73–82.

29. Silveira PCL, Silva LA, Tuon T, Freitas TP, Streck EL, Pinho RA. Efeitos da laserterapia de baixa potência na resposta oxidativa epidermica induzida pela cicatrização de feridas. Rev Bras Fisioter. 2009;13(4):281–7.

30. Kelner N, Castro J. Laser de baixa intensidade no tratamento da mucosite oral induzida pela radioterapia: relato de casos clínicos. Rev Bras Cancerol [Internet]. 2007;53(1):29–33. Available from: http://www1.inca.gov.br/rbc/n_53/v01/pdf/relato_caso1.pdf

31. Rodrigues, RN, Ferreira, VDP, Bittencourt, MC, Peixoto, IVP. Terapia com Laser de Baixa Intensidade na Cicatrização de Lesões por Pressão em Paciente Oriundo da UTI. Revista Científica Multidisciplinar Núcleo do Conhecimento. 2018; 03, 05(05):359-418.

32. Lima AAS de, Grégio AMT, Tanaka O, Machado MÂN, França BHS. Tratamento das ulcerações traumáticas bucais causadas por aparelhos ortodônticos. Rev Dent Press Ortod e Ortop Facial [Internet]. 2005;10(5):30–6. Available from: http://www.scielo.br/scielo.php?script=sci_arttext&pid=S1415-54192005000500005&lng=pt&tlng=pt

33. Luiza Zanette REOLONa, Lilian RIGOa*, Ferdinando de CONTOb LCC. Impacto da laserterapia na qualidade de vida de pacientes oncológicos portadores de mucosite oral. 2017;46(1):19–27.

34. Annaji S, Sarkar I, Rajan P, Pai J, Malagi S, Bharmappa R, et al.

Efficacy of photodynamic therapy and lasers as an adjunct to scaling and root planing in the treatment of aggressive periodontitis : A clinical and microbiologic short term study. J Clin Diagnostic Res. 2016;10(2):ZC08-ZC12.

35. Kajagar BM, Godhi AS, Pandit A, Khatri S. Efficacy of Low Level Laser Therapy on Wound Healing in Patients with Chronic Diabetic Foot Ulcers-A Randomised Control Trial. Indian J Surg. 2012;74(5):359–63.

36. Frozanfar A, Ramezani M, Rahpeyma A, Khajehahmadi S, Arbab HR. The effects of low level laser therapy on the expression of collagen type I gene and proliferation of human gingival fibroblasts (HGF3-PI 53): In vitro study. Iran J Basic Med Sci. 2013;16(10):1071–4.

37. Damante CA. Terapia com laser em baixa intensidade na cicatrização de feridas - revisão de literatura. Rfo. 2008;13(n.3):88–93.

38. Saúde EM. Brasil. Ministerio da Saúde. Secretaria de Vigilancia em Saúde. Departamento de Vigilancia Epidemiologica. Acidentes por Animais Peçonhnetos. In: Guia de vigilância em saúde. Ministério. Brasilia: Ministério da Saúde. Ms. 2014;1:719–38.

39. Núñez, SC. PROTOCOLO DE LASERTERAPIA E TERAPIA FOTODINÂMICA LASER DUO. 2ª ed. 2017. Disponível em: https://mmo.com.br/pt-br/protocolos/odontologia/file/239-protocolo-2-ed-laserterapia-e-tfp-laser-duo.

19. ATTACHMENTS

ATTACHMENT A

1. Patient Data

1.1 Phone contact: ( ) ______________ ( ) _________________
1.2 Address: ___________________________________n° ____ District: ___________
1.3 City: _________________
1.4 Gender: • 1-Male 2-Female
1.5 Birth date ____/____/____ **1.6** Age: ••

2. Snakebite Data
2.1 Origin: • 1-Manaus 2-Municipality Where? ____________________________

2.2 Date of the accident: ____/____/____

2.3 Estimate time of the accident:_____:_____

2.4 Date of admission to FMT-HVD:____/____/____ **2.5** Time of Admission:_____:_____

2.6 Date of discharge from FMT-HVD: ____/____/____
2.7 Reason: • 1-Discharge 2-Death 3-Transference
2.8 Zone of Occurrence: • 1-Rural 2-Urban 3-Peri-urban 9-Ignored
2.9 Place of bite: • 1-Head 2-Arm 3-Leg 4-Torso 5-Neck 6-Hand 7-Thigh 8-Forearm 9-Foot 10-Other (Specify):________________

2.10 Affected side of the body: • 1-Right 2-Left 3-Center
2.11 Stood or walked after the accident: • 1-No 2-Yes For how long? ____ min.
2.12 Accident related to work: • 1-No 2-Yes
2.13 Prior accident: • 1-No 2-Yes How many?_____
Date of the last accident: ____/_____ (mm/yyyy)

Signature and Stamp: ____

Laser Therapy				
	D1	D2	D3	D7
Energy (J)				
Dose (cm^2)				
Time				
Power				
Anatomic Points				
Place				

Thermography				
	D1	D2	D3	D7
Ambient temperature				
Body temperature				
Software				
Temperature scale/range				
Emissivity standard				
Anatomic Area				
Color/Temperature				

Strictly confidential

ATTACHMENT B

8. Patient's Clinical Data - Day 1

8.1 Date of assessment: I__I__II__I__II__I__I **8.2 Time**: ____:____

8.3 Blood pressure I__I__I__I / I__I__I__I mm Hg **8.4 Pulse** I__I__I__I min

8.5 Temperature I__I__I. I__I °C **8.6 Respiratory rate** I__I__I rpm

8.7 Concomitant complaints

- 1-No 2-Yes (Especify):____

8.8 Local Demonstrations: 1-No 2-Yes

8.8.1 **Bleeding**
- bleeding in the skin or mucous membranes
- evident ecchymosis
- bleeding without impairment of general condition

8.8.2 **Edema**
- Mild - local edema of up to 2 segments
- Moderate - local edema of 3 to 4 segments
- Severe - local edema of 5 segments

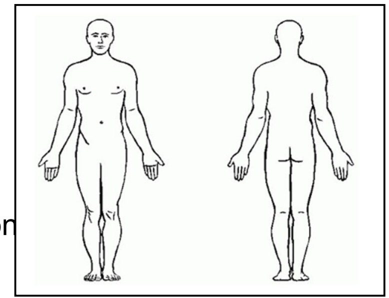

- Circumference of the affected region (centimeters)

Right side: ______cm Left side: _____cm Odd region

Extension of the edema: ______cm*

* mark the edema on the figure, both distal and proximal to the bite (mark the place of bite with the letter P)

8.8.3 **Lymph nodes** • Absent • Present Size (cm): ________

Consistency • elastic • stony • soften

Sensitivity • painful • painless

Mobility • attached to deep planes • not attached (mobile)

8.8.4 **Pain - Rating on a scale of 0 - 10**

Rate*: • Absent: 0 Mild: 1-3 Moderate: 4-7 Serious: 8-10

* After use of analgesic

8.8.5 **Perilesional Tonality:** 1-No 2-Yes
• normal • violet • redness • necrotic (blackened)

8.8.6 **Skin Characteristics:** 1-No 2-Yes
• Ecchymosis • Blisters • Serous secretion • Only the bite mark

9.8.7 Temperature: Affected area _____°C 9.8.8 Contralateral area _____°C

9. Systemic Manifestations: 1-No 2-Yes (mark with an X)
• Anuria • Headache • Shock •Abdominal cramps • Seizure
• Diarrhea • Enterorrhagia (melena and hematochezia) • Epistaxis
• Ecchymosis • Gingivorrhagia • Hematemesis • Hematuria
•Hemoptysis • Conjunctival hemorrhage • Hypermenorrhagia • Nausea
• Oliguria • Otorragia • Petechiae • Sweating • Vomit
• Others:____

Signature and Stamp: ____

ATTACHMENT C

Laboratory Tests

Blood

	DATA	D1	D2	D3	D4	D7	UN.	REF.
E R Y	HEMATIDS						millions/ mm^3	4.7 A 6.1
T H	HEMOGLOBIN						G/DL	13,0 A 16,0
R O	HEMATOCRIT						%	40,0 A 52,0
G	MCV						FL	80 A 97
R A	MCH						PG	27,0 A 31,0
M	MCHC						G/DL	33,0 A 37,0
	RDW						%	11,5 A 14,5%
	LEUKOCYTES						/ mm^3	4.000-10.800
L E	SEGMENTS						%/ mm^3	42,1 A 75,2
U	MYELOCYTES						%/ mm^3	
K O	METAMYELOCYTES						%/ mm^3	
G	BAND CELLS						%/ mm^3	
R	EOSINOPHILS						%/ mm^3	0,0 A 3,0
A	BASOPHILS						%/ mm^3	0,0 A 3,0
M	LYMPHOCYTES						/ mm^3	20,0 A 51,1
	MONOCYTES						/ mm^3	
P L	PLATELETS						/ mm^3	130.000-400.000
A T	MPV						FL	7,40 A 10,4
E	PCT							
L E T O G R A M	PDW							
C O	CT						MINUTES	UP TO 10
A	FIBRINOGEN						G/DL	2 to 4
G U	TAP						"/%	13.5"= 100%

LOGRAM	INR							1.0
INFLAMMATORY MARKERS	CK							
	CK-MB							
	REACTIVE C PROTEIN							
	ESR							
	LDH							
	AST							
	ALT							
