# Supplementary material for: Photobiomodulation Therapy to Treat Snakebites Caused by Bothrops atrox: A Randomized Clinical Trial
Source: JAMA Intern Med. 2023 Dec 4;184(1):70–80. doi: 10.1001/jamainternmed.2023.6538 (PMC10696517; doi:10.1001/jamainternmed.2023.6538)
Supplement: Supplement 2. — eFigure 1 eFigure 2 [file jamainternmed-e236538-s002.pdf]

Supplemental Online Content

Carvalho ÉS, Souza ARN, Melo DFC, et al. Photobiomodulation therapy to treat snakebites caused by *Bothrops atrox*: a randomized clinical trial. *JAMA Intern Med.* Published online December 4, 2023. doi:10.1001/jamainternmed.2023.6538

eFigure 1

eFigure 2

This supplemental material has been provided by the authors to give readers additional information about their work.

© 2023 Carvalho ÉS et al. *JAMA Internal Medicine.*

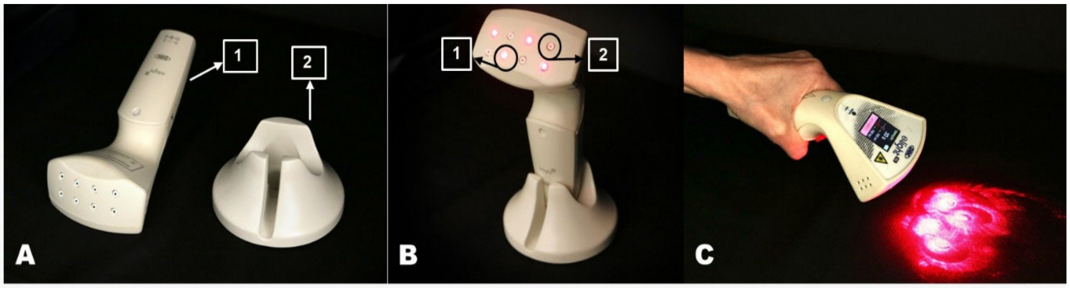

eFigure 1. A) 1. e-Light IRL (SN 0608) (DMC, São Carlos, Brazil) device used in the low-intensity laser therapy (LLLT); 2. e-Light IRL support base. B) 1. Red wave beam emission point; 2- Infrared wave beam. C) Combined view of light beams in a dark environment on a flat surface. Photo taken by the first author.

© 2023 Carvalho ÉS et al. *JAMA Internal Medicine.*

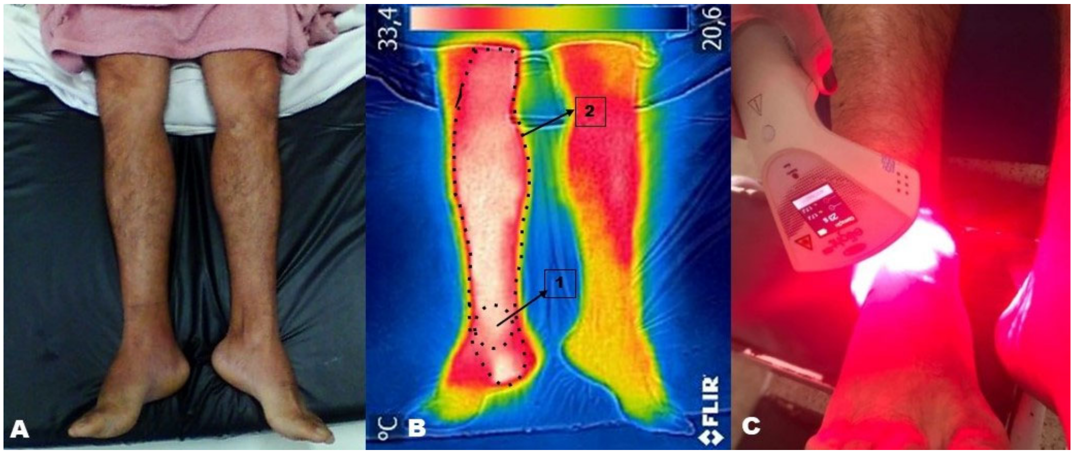

eFigure 2. A) Picture of a male patient diagnosed with a *Bothrops* snakebite, bitten in the lower right limb, on the internal aspect of the ankle, showing swelling and redness of the surrounding area, compared to the contralateral limb. B) Thermographic image of the same patient taken with a FLIR C2 thermographic camera (Teledyne FLIR, Sorocaba, Brazil). The surface of the limbs varies in color from dark blue, which is the coldest area, to white, which is the warmest area and which represents the region with the most intense inflammation. It is possible to observe that the white areas only appear on the bitten limb, in this case extending from the inner surface of the foot (below the fang marks in B.1) to a little below the knee (B.2). In this study, low-intensity laser therapy (LLLT) was applied to the white areas. C) LLLT procedure in the region previously identified with the help of thermography. Photo taken by the first author.

© 2023 Carvalho ÉS et al. *JAMA Internal Medicine.*
