# Supplementary material for: Photobiomodulation Therapy to Treat Snakebites Caused by Bothrops atrox: A Randomized Clinical Trial
Source: JAMA Intern Med. 2023 Dec 4;184(1):70–80. doi: 10.1001/jamainternmed.2023.6538 (PMC10696517; doi:10.1001/jamainternmed.2023.6538)
Supplement: Supplement 3. — Data Sharing Statement [file jamainternmed-e236538-s003.pdf]

Data Sharing Statement

Carvalho. Photobiomodulation Therapy to Treat Snakebites Caused by Bothrops atrox. *JAMA Intern Med*. Published December 04, 2023. doi:10.1001/jamainternmed.2023.6538

Data
Data available: No
